# A new caenagnathid dinosaur from the Upper Cretaceous Wangshi Group of Shandong, China, with comments on size variation among oviraptorosaurs

**DOI:** 10.1038/s41598-018-23252-2

**Published:** 2018-03-22

**Authors:** Yilun Yu, Kebai Wang, Shuqing Chen, Corwin Sullivan, Shuo Wang, Peiye Wang, Xing Xu

**Affiliations:** 1 Yuan Pei College, Peking University, Beijing, China; 2 Zhucheng Dinosaur Museum, Zhucheng, Shandong China; 3 Department of Biological Sciences, University of Alberta, Edmonton, Alberta T6G 2E9 Canada; 4 Philip J. Currie Dinosaur Museum, Wembley, Alberta T0H 3S0 Canada; 5 Laboratory of Vertebrate Evolution, College of Life Science, Capital Normal University, Beijing, 100048 China; 6 State Key Laboratory of Palaeobiology and Stratigraphy, Nanjing Institute of Geology and Palaeontology, Chinese Academy of Sciences, Nanjing, 210008 China; 7 Key Laboratory of Vertebrate Evolution and Human Origins, Institute of Vertebrate Paleontology and Paleoanthropology, Chinese Academy of Sciences, Beijing, China

## Abstract

The bone-beds of the Upper Cretaceous Wangshi Group in Zhucheng, Shandong, China are rich in fossil remains of the gigantic hadrosaurid *Shantungosaurus*. Here we report a new oviraptorosaur, *Anomalipes zhaoi* gen. et sp. nov., based on a recently collected specimen comprising a partial left hindlimb from the Kugou Locality in Zhucheng. This specimen’s systematic position was assessed by three numerical cladistic analyses based on recently published theropod phylogenetic datasets, with the inclusion of several new characters. *Anomalipes zhaoi* differs from other known caenagnathids in having a unique combination of features: femoral head anteroposteriorly narrow and with significant posterior orientation; accessory trochanter low and confluent with lesser trochanter; lateral ridge present on femoral lateral surface; weak fourth trochanter present; metatarsal III with triangular proximal articular surface, prominent anterior flange near proximal end, highly asymmetrical hemicondyles, and longitudinal groove on distal articular surface; and ungual of pedal digit II with lateral collateral groove deeper and more dorsally located than medial groove. The holotype of *Anomalipes zhaoi* is smaller than is typical for Caenagnathidae but larger than is typical for the other major oviraptorosaurian subclade, Oviraptoridae. Size comparisons among oviraptorisaurians show that the Caenagnathidae vary much more widely in size than the Oviraptoridae.

## Introduction

Oviraptorosauria is a clade of maniraptoran theropod dinosaurs characterized by a short, high skull, long neck and short tail. Derived oviraptorosaurs can be divided into two groups: the Caenagnathidae and the Oviraptoridae. Most known caenagnathid taxa, including *Anzu wyliei*^1^, *Caenagnathus collinsi*^2^, *Chirostenotes pergracilis*^3^, *Apatoraptor pennatus*^4^ and *Microvenator celer*^5^, were collected from North America. However, several caenagnathids have been found in Asia, including *Caenagnathasia martinsoni*^6^, *Elmisaurus rarus*^7^, and the enigmatic *Gigantoraptor erlianensis*^1,8^.

**Figure 1:**
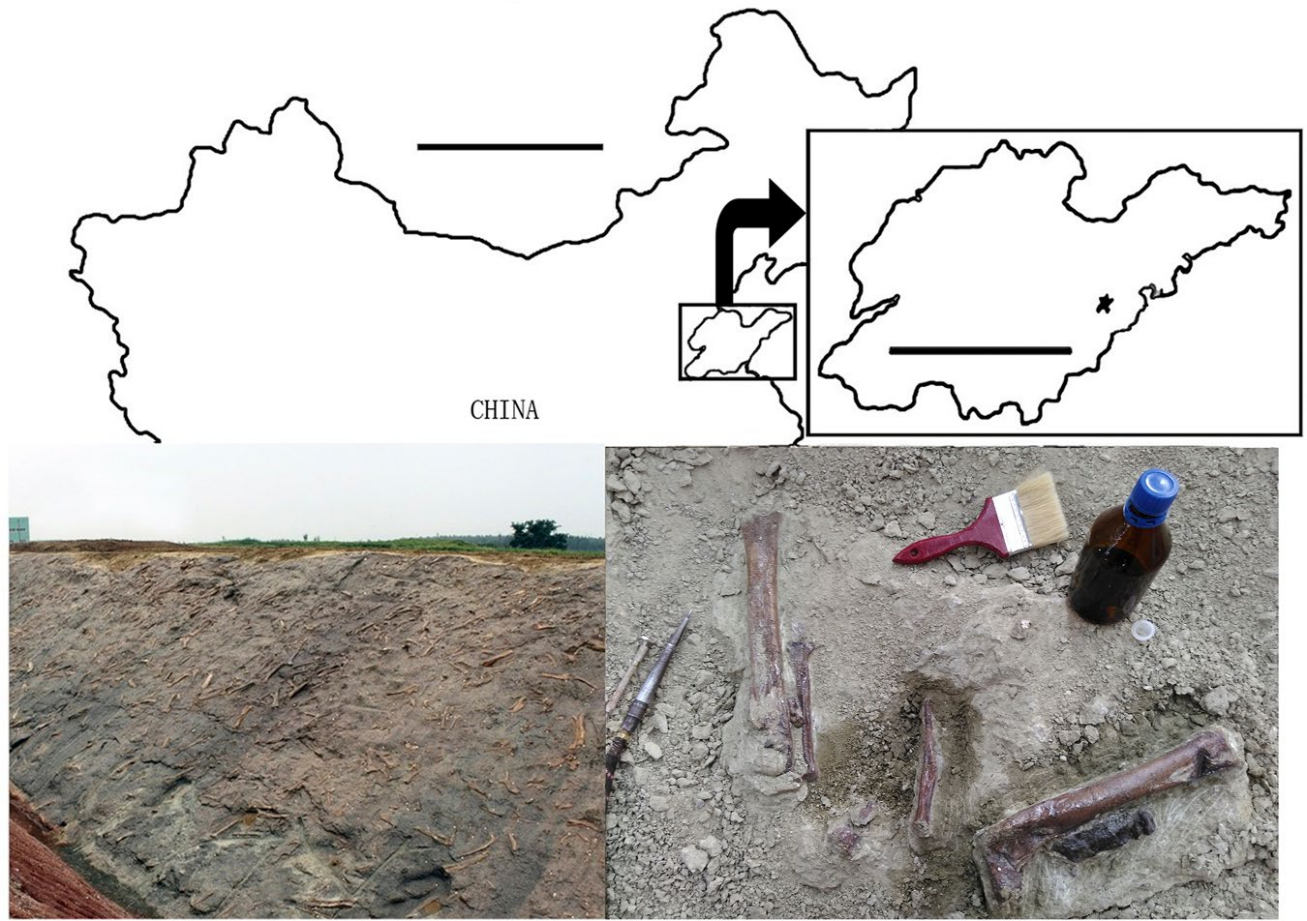
The type locality for *Anomalipes zhaoi* ZCDM V0020. Upper: map showing Zhucheng, Shandong Province, China (scale bars are 1000 km for China and 250 km for Shandong Province; star represents Zhucheng). Lower left: bone-bed exposed at the Kugou Locality. Lower right: holotype exposed during excavation. The map was traced by Yilun Yu in Photoshop CS6 (www.adobe.com/photoshop), and modified from Hone *et al*.^13^.

Over the last decade we have organized several major excavations in the Upper Cretaceous Wangshi Group at the Zangjiazhuang, Longgujian, and Kugou localities, Zhucheng, Shandong Province, China, which are in close geographic and stratigraphic proximity to each other, and produce similar dinosaur assemblages dominated by colossal hadrosaurid specimens. These excavations have yielded at least four new dinosaur taxa, including a tyrannosaurid, two leptoceratopsids, and a ceratopsid^9–13^. The tyrannosaurid, *Zhuchengtyrannus magnus*, represents the only documented theropod in the Zhucheng dinosaurian fauna, although some undiagnostic additional tyrannosaurid specimens were described previously under the invalid name *Tyrannosaurus zhuchengensis*^14^. Here we describe a new theropod specimen from the Kugou Locality (Fig. 1). The specimen comprises only a partial left hindlimb, but nevertheless displays a unique combination of morphological features suggesting the presence of a previously unknown caenagnathid oviraptorosaur in the Zhucheng dinosaurian fauna. This new discovery, along with previously reported fossil remains, provides strong evidence supporting a close biogeographical relationship between the Zhucheng dinosaurian fauna and contemporary North American ones.

## Results

### Systematic palaeontology

Theropoda Marsh 1881

Oviraptorosauria Barsbold 1976

Caenagnathidae Sternberg 1940

*Anomalipes zhaoi* gen.et sp. nov

#### Etymology

Generic name is a combination of the Latin “Anomalus” and “pes”, referring to the unusual shape of the foot. Specific name is in honour of Xijin Zhao, a Chinese palaeontologist who has made great contributions to research on Zhucheng dinosaur fossils.

#### Holotype

ZCDM V0020 (Zhucheng Dinosaur Museum, Zhucheng, Shandong, China), an incomplete left hindlimb, including the left femur missing the distal end, the left tibia missing the proximal end, the left fibula missing the distal and proximal ends, a complete metatarsal III and two pedal phalanges. Although these bones are disarticulated, they are inferred to be derived from a single theropod individual given that 1) they were preserved in a small area of less than 0.3 square metres within a *Shantungosaurus* bonebed; and 2) no other theropod skeletal elements are preserved nearby.

#### Locality and horizon

Kugou, Zhucheng City, Shandong Province, China. Upper Cretaceous Wangshi Group.

#### Diagnosis

A new caenagnathid with the following unique combination of features: femoral head anteroposteriorly narrow and somewhat deflected posteriorly; accessory trochanter low; lateral ridge present on femoral lateral surface; weak fourth trochanter present; metatarsal III with triangular proximal articular surface, prominent anterior flange near proximal end, medial hemicondyle much narrower than lateral hemicondyle, and longitudinal groove on distal articular surface; and pedal phalanx II-3 with lateral collateral groove deeper and more dorsally located than medial groove.

### Description and comparisons

The left femur is well preserved, lacking only its distal end. The preserved part of the shaft is 277 mm long. Comparisons with the distal portions of the femora of other oviraptorosaurs (e.g. *Nomingia gobiensis*)^15^, based on such features as the presence of an intercondylar groove on the posterior surface of the distal portion of the preserved femoral shaft, suggest that the original length of the femur must have been approximately 300 mm. The femoral shaft is straight in anterior view and only slightly bowed anteriorly in lateral view, as in other derived Oviraptorosauria^1,8,16–20^.

**Figure 2:**
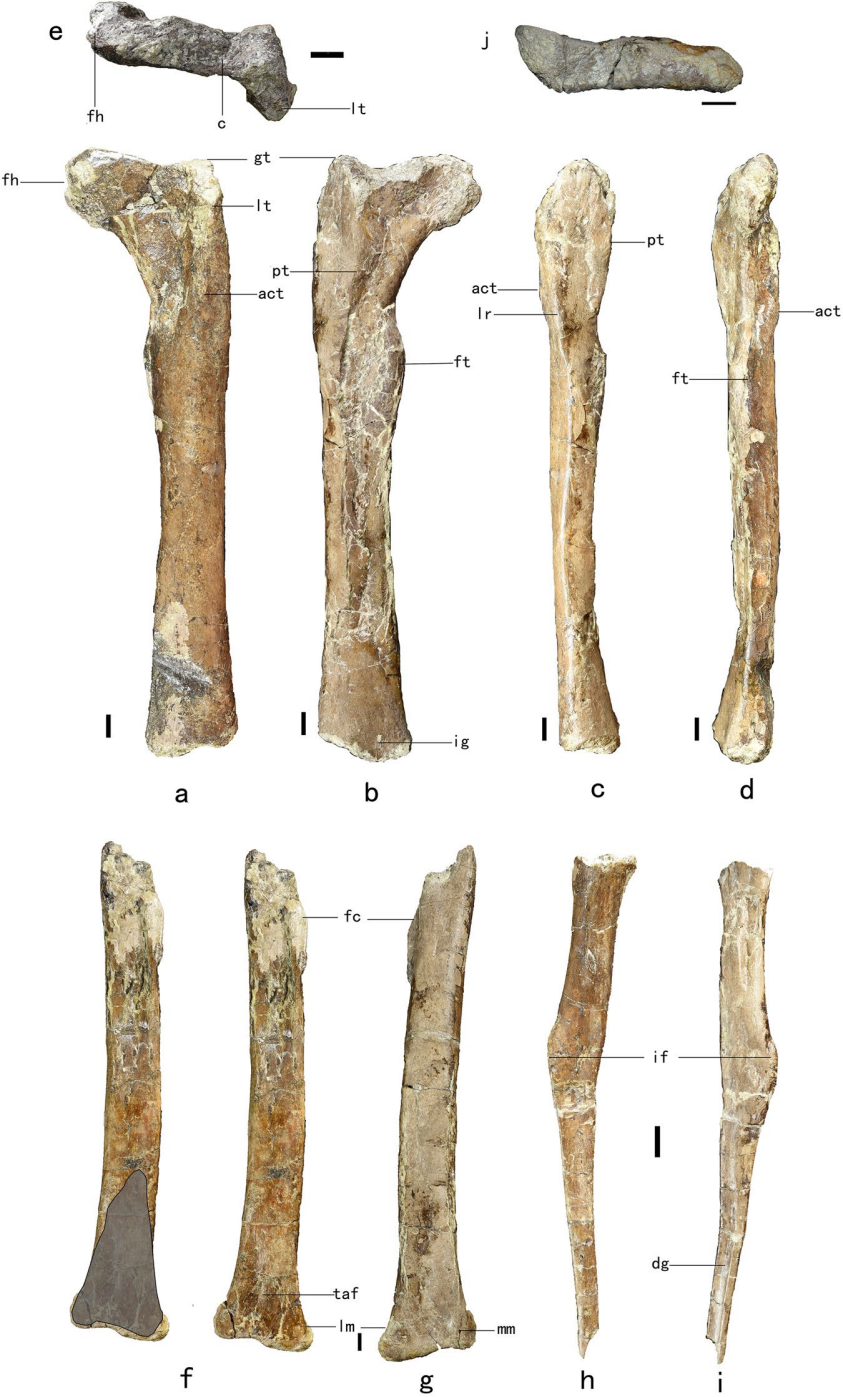
Preserved left femur, tibia, and fibula of *Anomalipes zhaoi* ZCDM V0020. Left femur in anterior (**a**), posterior (**b**), lateral (**c**), medial (**d**) and proximal (**e**) views. Left tibia in anterior (**f**), posterior (**g**) and distal (**j**) views. Shading indicates the articular facet for the ascending process of the astragalus. Left fibula in lateral (**h**) and medial (**i**) views. Abbreviations: act, accessory trochanter; dg, distinct groove; fc, fibular crest; fh, femoral head; ft, fourth trochanter; gt, greater trochanter; if, iliofibularis tubercle; ig, intercondylar groove; lm, lateral malleolus; lr, lateral ridge; lt, lesser trochanter; mm, medial malleolus; pt, posterior trochanter; taf, triangular articular facet. Scale bar 1 cm.

The femoral head is anteroposteriorly narrow, unlike its more robust counterparts in other oviraptorosaurs^1,4–6,8,17,18,21,22^. It projects dorsomedially, forming an angle of 100° with the long axis of the shaft in anterior view, and also is strongly posteriorly deflected as in *Gigantoraptor erlianensis*^8^. The shape and orientation of the femoral head are almost certainly morphologically genuine because the femur in general has undergone little deformation during fossilization, though the posterior surface of the middle portion of the shaft is crushed inward. A circular fovea seems to be present on the medial surface of the femoral head (Fig. 2d), but this might be a preservational artefact. There is a wide oblique ligament sulcus on the posterior surface of the femoral head. A wide concavity separates the femoral head from the trochanteric crest to define a distinct femoral neck, which is also slightly constricted in proximal view (Fig. 2e) as in *Anzu wyliei*^1^, *Chirostenotes pergracilis*^3^, *Caenagnathasia martinsoni*^6^, *Nankangia jiangxiensis*^18^, *Caenagnathus collinsi*^2^, *Khaan mckennai*^17^, *Gigantoraptor erlianensis*^8^, *Ajancingenia yanshini*^19^, *Microvenator celer*^5^, *Nomingia gobiensis*^15^ and *Avimimus portentosus*^21^. The ventral margin of the femoral neck is straight in anterior view as in *Caenagnathasia martinsoni*^6^, rather than curved as in *Chirostenotes pergracilis*^3^, *Khaan mckennai*^17^, *Nankangia jiangxiensis*^18^, *Anzu wyliei*^1^ and *Caenagnathus collinsi*^2^ (Fig. 2a). The greater trochanter is anteroposteriorly expanded and has fused at least partially with the lesser trochanter and posterior trochanter to form a trochanteric crest, which is anteroposteriorly much wider than the femoral head as in other oviraptorosaurs^1,3,5,6,8,15,17–19,21^. The trochanteric crest is thicker and higher anteriorly than posteriorly, as in *Gigantoraptor erlianensis*^8^. However, the trochanteric crest is lower than the femoral head, due to the dorsal inclination of the latter. The posterior trochanter is prominent as in *Khaan mckennai*^17^, *Nankangia jiangxiensis*^18^, *Ajancingenia yanshini*^19^ and *Gigantoraptor erlianensis*^8^, whereas this structure is more subtly developed in *Anzu wyliei*^1^ and *Caenagnathasia martinsoni*^6^. The lesser trochanter is probably fully continuous with the greater trochanter, but this needs further confirmation because the proximal end of the lesser trochanter is broken. Nevertheless, the lesser trochanter is unlikely to be a broad wing-like structure deeply separated from the greater trochanter as in most non-pennaraptoran theropods, except derived therizinosauroids^22^ and derived alvarezsauroids^23^, but is probably cylindrical in cross section as in other pennaraptorans.

An accessory trochanter is present at the base of the lesser trochanter, and extends distally to a level about one-fourth of the way to the distal end of the femur (Fig. 2a,c,d). A prominent accessory trochanter has also been reported in the basal oviraptorosaurs *Caudipteryx zoui*^24^ and *Avimimus portentosus*^21^, and in several caenagnathids including *Microvenator celer*^5^, *Caenagnathus collinsi*^2^, *Anzu wyliei*^1^ and *Chirostenotes pergracilis*^3^. Posterior to the accessory trochanter, on the lateral surface of the femur, is situated a thick lateral ridge that extends further distally than the accessory trochanter (Fig. 2c). Such a ridge is not known in other oviraptorosaurs^1,2,5,6,8,15,17,18^, but is present in troodontids^25,26^ and dromaeosaurids^27–29^. The proximal end of this lateral ridge forms a slight expansion, which might represent a weak trochanteric shelf (posterolateral trochanter of some authors). Unlike in most oviraptorosaurs, but as in *Avimimus portentosus*^21^ and *Caenagnathasia martinsoni*^6^, a fourth trochanter is present along the posteromedial margin of the femoral shaft. Although the distal end of the femur is broken away, the intercondylar groove is continuous with a depression on the distalmost preserved portion of the posterior surface, which is probably a part of the popliteal fossa.

The left tibia is 325 mm long as preserved, and only the proximal end is missing. Comparisons with the proximal portions of the tibiae of other oviraptorosaurians (e.g. *Nomingia gobiensis*)^15^, based on such features as the relative position of the fibular crest, suggest that the original length of the tibia was about 360 mm. Combined with the estimated femoral length of 300 mm given earlier, this measurement would suggest a tibial length to femoral length ratio of approximately 1.20, similar to the value for *Chirostenotes pergracilis*^3^ but lower than those for *Anzu wyliei*^1^, *Microvenator celer*^5^, *Caudipteryx zoui*^24^, and *Nomingia gobiensis*^15^. The tibial shaft is slightly curved in anterior view, being convex in the lateral direction. The shaft is much greater in mediolateral width than in anteroposterior thickness, and the proximal half of the shaft has a sub-semilunate cross section due to the convexity of the posterior surface and relative flatness of the anterior surface. A semi-circular cross section seems to characterize the oviraptorosaurian tibia (Gregory F. Funston, personal communications). A 47-mm-long, straight-edged fibular crest is located on the lateral surface of the shaft, and does not continue to the proximal end of the tibia (Fig. 2f,g). The fibular crest trends slightly anteriorly as it extends distally. The transverse width of the tibial distal end is greater than that of the midshaft part of the bone. Immediately proximal to the distal end, the anterior surface of the tibial shaft bears a distinct large, triangular articular facet to accommodate the ascending process of the astragalus. This facet is proximodistally tall, and shifted laterally from the midline of the anterior surface of the shaft (Fig. 2f). The lateral and medial malleoli extend distally to nearly the same level (Fig. 2f,g), and a similar condition is present in *Anzu wyliei*^1^*, Chirostenotes pergracilis*^3^*, Caudipteryx zoui*^24^ and the Oviraptoridae. The distal surface of the tibia is sub-rectangular (Fig. 2j).

The left fibula is missing the proximal articular surface and the distal portion, and has a preserved length of 170 mm. The proximal portion of the fibula is somewhat D-shaped in cross section, with a convex lateral surface and a flat medial surface. A prominent iliofibularis tubercle, which is about 20 mm long proximodistally, projects anterolaterally from the shaft (Fig. 2h,i). Distal to the iliofibularis tubercle, the fibular shaft gradually tapers. The medial surface of the distalmost portion of the fibular shaft bears a distinct groove.

**Figure 3:**
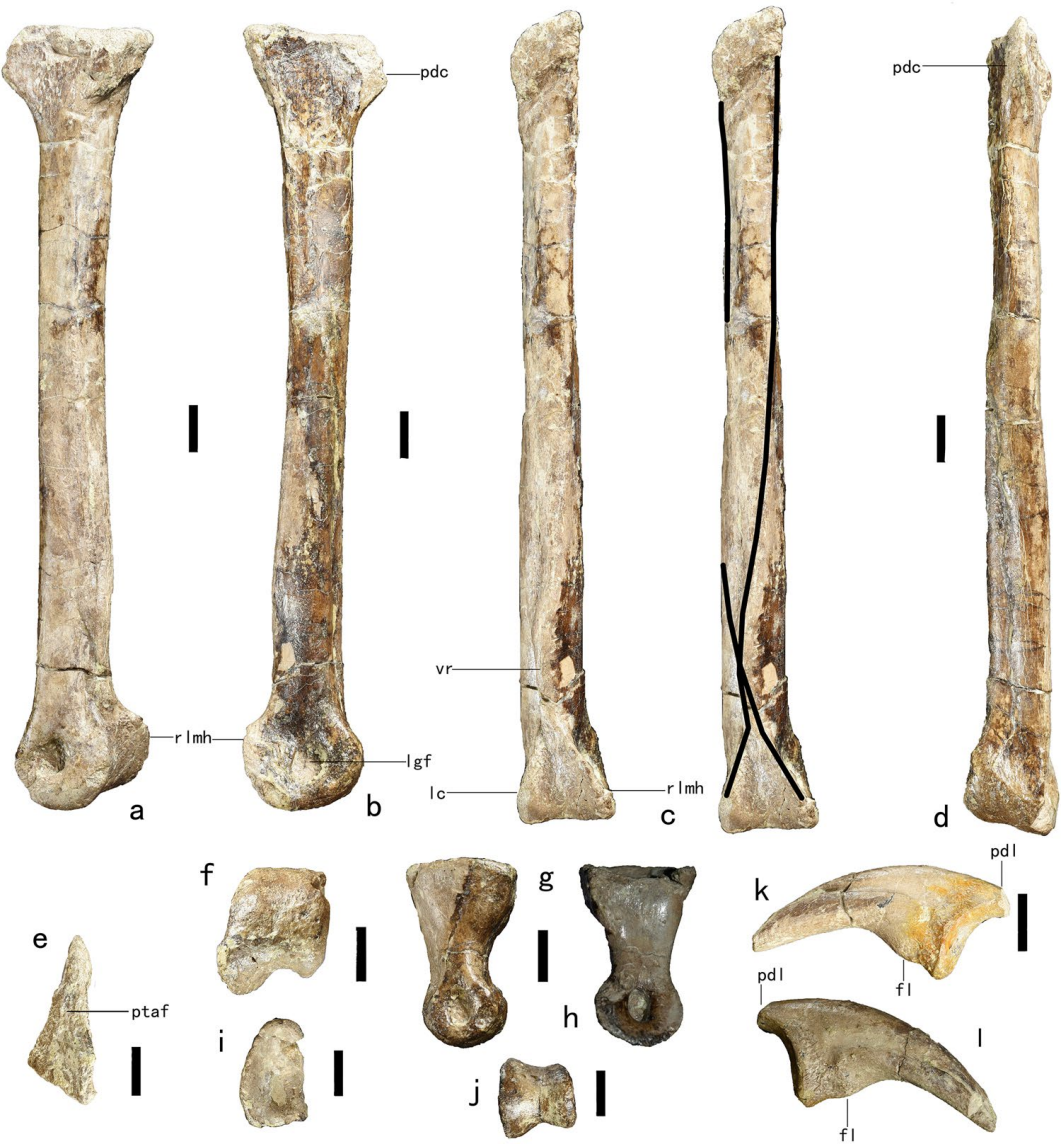
Preserved pedal elements of *Anomalipes zhaoi* ZCDM V0020. Left metatarsal III in lateral (**a**), medial (**b**), posterior (**c**), anterior (**d**), proximal (**e**) and distal (**f**) views. Dark lines indicate ridges on the posterior surface of the shaft. Phalanx IV-1 in lateral (**g**), medial (**h**), proximal (**i**), and distal (**j**) views. Phalanx II-3 in lateral (**k**) and medial (**l**) views. Abbreviations: fl, flexor tubercle; lc, lateral condyle; lgf, ligament fossa; pdc, proximal dorsal crest; pdl, proximal dorsal lip; vr, ventral ridge; ptaf, proximal triangular articular facet; rlmh, ridge-like medial hemicondyle; vr, ventral ridge (extending to medial hemicondyle). Scale bar 1 cm.

The left metatarsal III is completely preserved (Fig. 3a–f), with a measured length of 167 mm. Based on the estimated femoral length of 300 mm, the ratio of the length of metatarsal III to that of the femur is 0.56, a value comparable to those for most caenagnathids but greater than those for most oviraptorids. The proximal end of metatarsal III is transversely strongly compressed, the ratio of maximum anteroposterior thickness to maximum transverse width being about 2.1. The compression is greatest at the anterior margin of the proximal end, so that the proximal articular surface of metatarsal III is triangular in outline (Fig. 3e). A transversely pinched proximal end of metatarsal III is also seen in other caenagnathids and in some basal oviraptorosaurs^3,7,21,24^, and in some cases (e.g. *Chirostenotes pergracilis, Elmisaurus rarus* and *Avimimus portentosus*) the compression is sufficiently extreme to produce an arctometatarsalian pes. However, the more distal portion of the shaft is transversely much wider than anteroposteriorly deep in some caenagnathids^30,31^, as in some theropods with a proximally pinched metatarsal III such as the troodontid *Sinovenator* (IVPP V12583). The compressed proximal portion of the anterior surface of metatarsal III forms a prominent flange (Fig. 3a,b,d). The proximalmost part of the medial surface of the shaft bears a triangular articular facet for metatarsal II. The shaft of metatarsal III is anteroposteriorly deeper than transversely wide, and has a sub-rectangular cross-section proximally and a triangular one more distally. *Gigantoraptor erlianensis* has a similar metatarsal III: the proximal end is about twice as anteroposteriorly deep as transversely wide; an anterior flange, albeit a proximodistally shorter one than in *Anomalipes zhaoi*, is present; and the shaft is sub-rectangular proximally (though slightly transversely wider than anteroposteriorly deep) and sub-triangular distally in cross section^8^. The posterior surface of the shaft bears a longitudinal ridge, whose proximal half is situated along the posteromedial edge of the shaft but whose distal half curves laterally as it extends distally. Near the distal end of the metatarsal, the ridge meets centrally along the shaft with the proximolaterally oriented medial hemicondyle (Fig. 3c). A second ridge, albeit an extremely weak one, runs along the posterolateral edge of the proximal part of the shaft but terminates about halfway along the shaft’s length. The distal articular surface of metatarsal III is slightly ginglymoid (Fig. 3c,d). The distal end of metatarsal III is more deeply grooved in *Gigantoraptor erlianensis* than in the present specimen^8^, but is non-ginglymoid in all other oviraptorosaurs. The anteroposterior thickness of the distal end is about 1.2 times the mediolateral width, and the anterior margin of the distal end is narrower than the posterior margin in distal view. The posterior hemicondyles of the distal end take the form of thick ridges and are both oriented obliquely, with the medial one extending proximolaterally and the lateral one proximomedially. The condyles converge on the central part of the shaft as they extend proximally, a feature also seen in some other caenagnathids^30,31^. The medial condyle is transversely wider and much more prominent in the posterior direction than the lateral condyle. A prominent, oblique, ridge-like medial hemicondyle is also seen at the distal end of metatarsal III in *Gigantoraptor erlianensis*^8^ and a similar, but much less strongly developed, medial hemicondyle is probably present in many other caenagnathids^30,31^. In *Elmisaurus rarus*^7^, *Chirostenotes pergracilis*^3^, *Avimimus portentosus*^21^ and *Elmisaurus elegans*^16^, the medial hemicondyle is slightly larger than the lateral one. Both medial and lateral collateral ligament fossae are present, and are large and deep (Fig. 3f).

Two left pedal phalanges are preserved, including one proximal phalanx and one ungual. The proximal phalanx, which probably represents pedal phalanx IV-1, is robust, the proximodistal length of the bone being only about 1.8 times the transverse width at the proximal end (Fig. 3g,h). The proximal articular surface is a deep concavity (Fig. 3i). In dorsal view, the mid-length portion of the shaft is mediolaterally constricted, and there is a moderately well developed extensor fossa close to the distal end. In medial view, the proximal half of the ventral surface of the phalanx can be seen to protrude ventrally as a prominent, block-like heel. The lateral surface of the shaft is convex along its length, and the medial surface is slightly concave. The distal end is strongly ginglymoid, with the medial hemicondyle larger than the lateral hemicondyle (Fig. 3j). The medial collateral ligament fossa is deeper than the lateral one (Fig. 3g,h).

The preserved ungual, possibly that of digit II, is robust and moderately recurved ventrally (Fig. 3k,l). When the ungual is held so that the proximal articular facet is vertical, the tip of the ungual is directed nearly ventrally. The proximal articular surface is sub-oval in outline, with a vertical ridge at the midline. Close to the proximal end is an extremely weak flexor tubercle, which is proximally bounded by ventral branches of the collateral groove on both the medial and the lateral sides. There is a proximodorsal lip, but the original size of this feature is uncertain due to breakage. The lateral surface of the ungual is more convex than the medial surface. Collateral grooves are present on both the medial and lateral surfaces of the ungual, and they each bifurcate proximally into a dorsal branch extending close to the proximodorsal corner of the ungual and a ventral branch extending to the ventral margin near the proximal end. The collateral grooves are proximally wide and shallow, but distally narrow and deep. The lateral collateral groove is deeper and more dorsally positioned than the medial groove, and its distal portion nearly contacts the ungual’s dorsal margin.

## Methods

### Phylogenetic analyses

**Figure 4:**
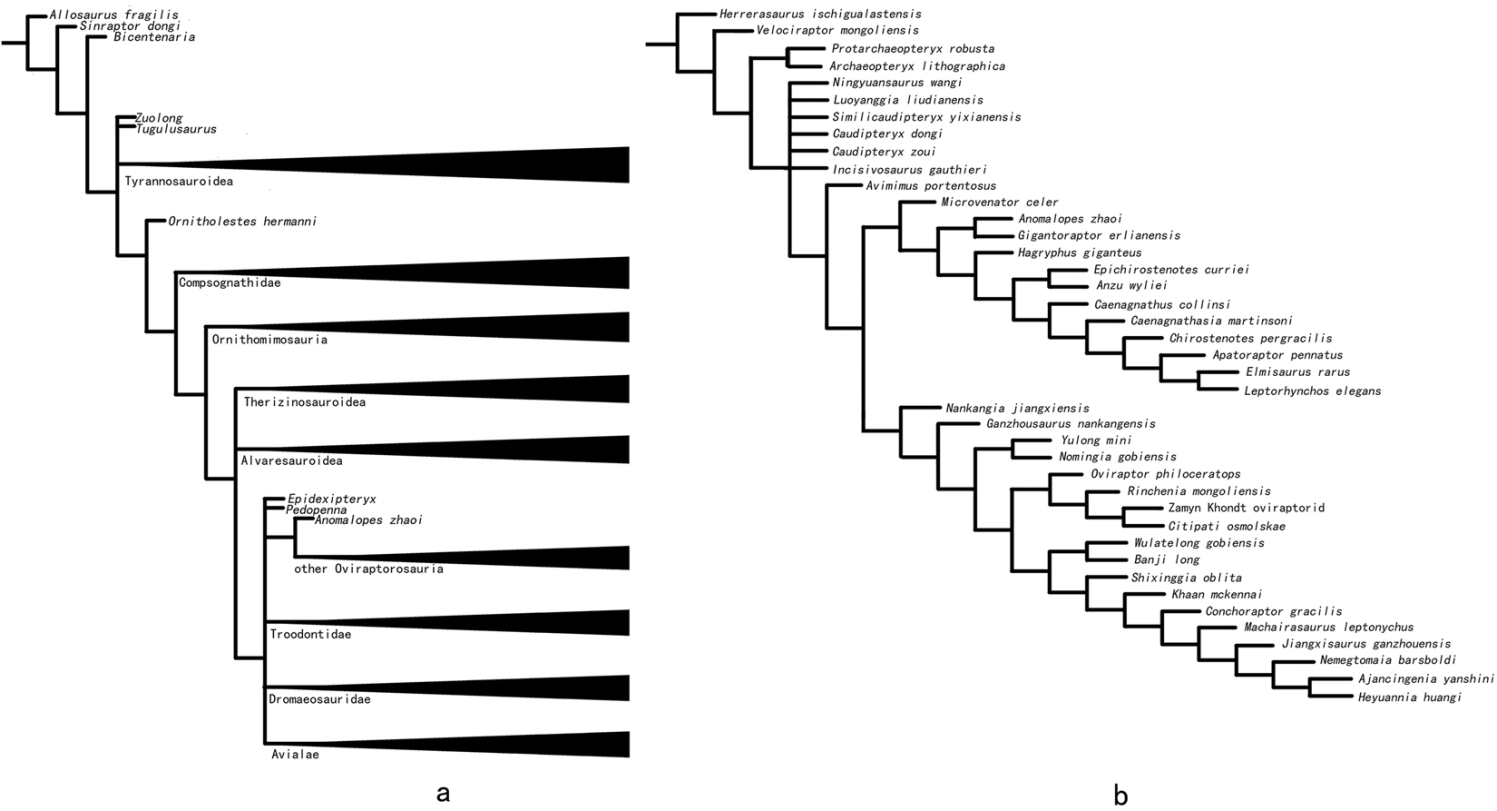
Systematic position of *Anomalipes zhaoi* among the Coelurosauria. (**a**), Simplified reduced strict consensus of 73 most parsimonious trees resulting from phylogenetic analyses of dataset derived from Brusatte *et al*. (2014), with *Anomalipes zhaoi* and 13 new characters added in. (**b**), Simplified strict consensus of 3 most parsimonious trees resulting from phylogenetic analysis of dataset derived from Funston and Currie (2016), with 14 new characters and *Anomalipes zhaoi* added in.

In order to determine the systematic position of *Anomalipes zhaoi*, we conducted a phylogenetic analysis of a matrix derived from a recently published comprehensive dataset for coelurosaurian theropod phylogeny^32^, but with the addition of *Anomalipes zhaoi* and 13 new characters (see Supplementary Information). The matrix was analysed using a “New technology search” in TNT version 1.1. Default settings were used for most parameters, but the value for “Find min. length” was changed from 1 to 10. The analysis produced 73 most parsimonious trees, each with a tree length of 3516, a CI of 0.311, and an RI of 0.775. Because the strict consensus of these 73 trees was poorly resolved in several areas, we calculated their reduced consensus following a previous study^32^. The reduced consensus is shown in Fig. 4a, and places *Anomalipes zhaoi* within the Oviraptorosauria.

We performed additional phylogenetic analyses in order to more precisely investigate the position of *Anomalipes zhaoi* within the Oviraptorosauria. The analyses were based on two recently published datasets for evaluating oviraptorosaurian phylogeny, respectively compiled by^1^ and Funston and Currie (2016), but the datasets were used with some modifications (see Supplementary Information). The two matrices (here termed the Lamanna Matrix and Funston and Currie Matrix, respectively) were analyzed in TNT version 1.1, using a traditional search (1000 replicates, 1000 random seeds, 10 trees saved per replication). Analysis of the Lamanna Matrix produced 860 most parsimonious trees, each with a tree length of 552, a CI of 0.524 and a RI of 0.687. Analysis of the Funston and Currie Matrix produced 3 most parsimonious trees, each with a tree length of 620, a CI of 0.497 and a RI of 0.675. Both analyses posited *Anomalipes zhaoi* as the sister taxon to *Gigantoraptor erlianensis*^8^ within the Caenagnathidae, and Fig. 4b shows the strict consensus of the most parsimonious trees produced by the analysis of the Funston and Currie Matrix (Fig. 4b).

## Discussion

*Anomalipes zhaoi* is clearly a maniraptoran theropod, based on the presence of some distinctive maniraptoran hindlimb features. A distinct femoral neck, defined dorsally by an anteroposteriorly oriented concavity, separates the greater trochanter from the femoral head. This feature is seen in Oviraptorosauria, Therizinosauroidea^33–35^, Troodontidae^36–38^, and Alvarezsauroidea^39,40^. A similar groove is absent in basalmost theropods^41^, ceratosaurs^42^, basal tetanurans^43–45^, tyrannosauroids^46–49^, and ornithomimosaurs^50–52^. The greater trochanter is also expanded anteroposteriorly to the point of being considerably wider than the femoral head, as in Oviraptorosauria^1,3–6,8,17,18,21,24,53^, Troodontidae^26,36–38^, Dromaeosauridae^27,28,54,55^, and Therizinosauroidea^33–35^. In more basal theropods, the greater trochanter is anteroposteriorly narrower than the femoral head^45,48–50^. The distal end of the tibia has a sub-rectangular outline in distal view, resembling the equivalent structure in many other maniraptorans; in non-maniraptoran theropods such as ornithomimosaurs, tyrannosaurids, sinraptorids, carcharodontosaurids and abelisaurids, the distal articular surface is sub-triangular in distal view^56^.

In the context of Maniraptora, *Anomalipes zhaoi* displays several features suggesting pennaraptoran affinities. For example, the femoral lesser trochanter is finger-shaped and adheres to the greater trochanter. This stands in stark contrast to the condition in most non-pennaraptoran theropods, in which the lesser trochanter is separated from the greater trochanter by a deep cleft and is either alariform or spike-like. The only known non-pennaraptoran theropods with a finger-shaped lesser trochanter closely adhering to the greater trochanter are derived alvarezsauroids^23,39^, which are consistently much smaller than *Anomalipes zhaoi*, and derived therizinosauroids^22^, which dramatically differ from *Anomalipes zhaoi* in other hindlimb features.

Among the major pennaraptoran groups, *Anomalipes zhaoi* shows the clearest morphological resemblances to the Oviraptorosauria. For example, an elongate accessory trochanter is present, as in many basal oviraptorosaurs and caenagnathids. An accessory trochanter is not known in troodontids or in most dromaeosaurids, although this feature occurs in some small microraptorines. *Anomalipes zhaoi* has a small fourth trochanter as in the oviraptorosaurians *Caenagnathasia martinsoni*^6^ and *Avimimus portentosus*^21^. A fourth trochanter is absent in troodontids and many dromaeosaurids, though several dromaeosaurids such as *Velociraptor mongoliensis* do have an extremely small fourth trochanter^28^. A prominent mound-like trochanteric shelf is widely present in maniraptorans, including troodontids and dromaeosaurids, but is absent in *Anomalipes zhaoi* as in derived oviraptorosaurs.

The limited material, and particularly the lack of cranial material, in the holotype and only known specimen of *Anomalipes zhaoi* makes it difficult to identify striking caenagnathid features in this species. However, some recent studies provide significant new information on caenagnathid hindlimb morphology^1,4,30,31^, which can be used to confirm the caenagnathid affinities of *Anomalipes zhaoi*. In general, caenagnathids possess an elongated hindlimb with a particularly long tibia, a fused astragalocalcaneum, coossified distal tarsals and metatarsals, and an arctometatarsalian foot with a proximally strongly pinched metatarsal III^1,31^. In *Anomalipes zhaoi* the lower segments of the hindlimb appear relatively elongated (e.g. the tibia is about 1.2 and 2.1 times as long as the femur and metatarsal III, respectively, based on length estimates for the femur and tibia given above; the ratio of metatarsal III length to estimated femur length is 0.56), more closely resembling the condition in caenagnathids than that in oviraptorids. *Anomalipes zhaoi* lacks a typical arctometatarsalian foot, but its metatarsal III has a transversely strongly compressed proximal end, suggesting that the foot was incipiently arctometatarsalian. The compactness of the foot in *Anomalipes zhaoi*, which is typical of caenagnathids but not of oviraptorids, is further indicated by the relative slenderness of metatarsal III, which displays a ratio of length to midshaft transverse width of about 14.0. The *Anomalipes zhaoi* hindlimb lacks some fusion features normally seen in caenagnathids, but this is also true of a few previously described caenagnathids such as *Gigantoraptor erlianensis*. Furthermore, a prominent accessory trochanter and weak fourth trochanter are features widely present in basal oviraptorosaurians and caenagnathids, but not in oviraptorids. Most strikingly, *Anomalipes zhaoi* seems to possess cruciate ridges on the posterior surface of metatarsal III, a feature that has been recently identified to be unique to some caenagnathids^30^, in incipient form. In general, the hindlimb morphology of *Anomalipes zhaoi* is consistent with and suggestive of a phylogenetic position among basal caenagnathids.

The fact that phylogenetic analysis of both the Lamanna Matrix and the Funston and Currie Matrix recovered *Anomalipes zhaoi* and *Gigantoraptor erlianensis* as an endemic Asian oviraptorosaurian clade deserves special note. Despite their very different sizes, *Anomalipes zhaoi* and *Gigantoraptor erlianensis* share many similarities, including several that are unique among the Oviraptorosauria. These shared unique features include: femoral head with strong posterior deflection; trochanteric crest thicker and higher anteriorly than posteriorly; posterior trochanter prominent; and metatarsal III with anterior flange near proximal end, longitudinal groove on distal articular surface, and prominent oblique, ridge-like medial hemicondyle at distal end. To determine whether these hindlimb features imply any locomotor peculiarities would require a strict biomechanical analysis, but they nevertheless indicate the presence of an unusual endemic oviraptorosaurian group in the Late Cretaceous of Asia.

**Figure 5:**
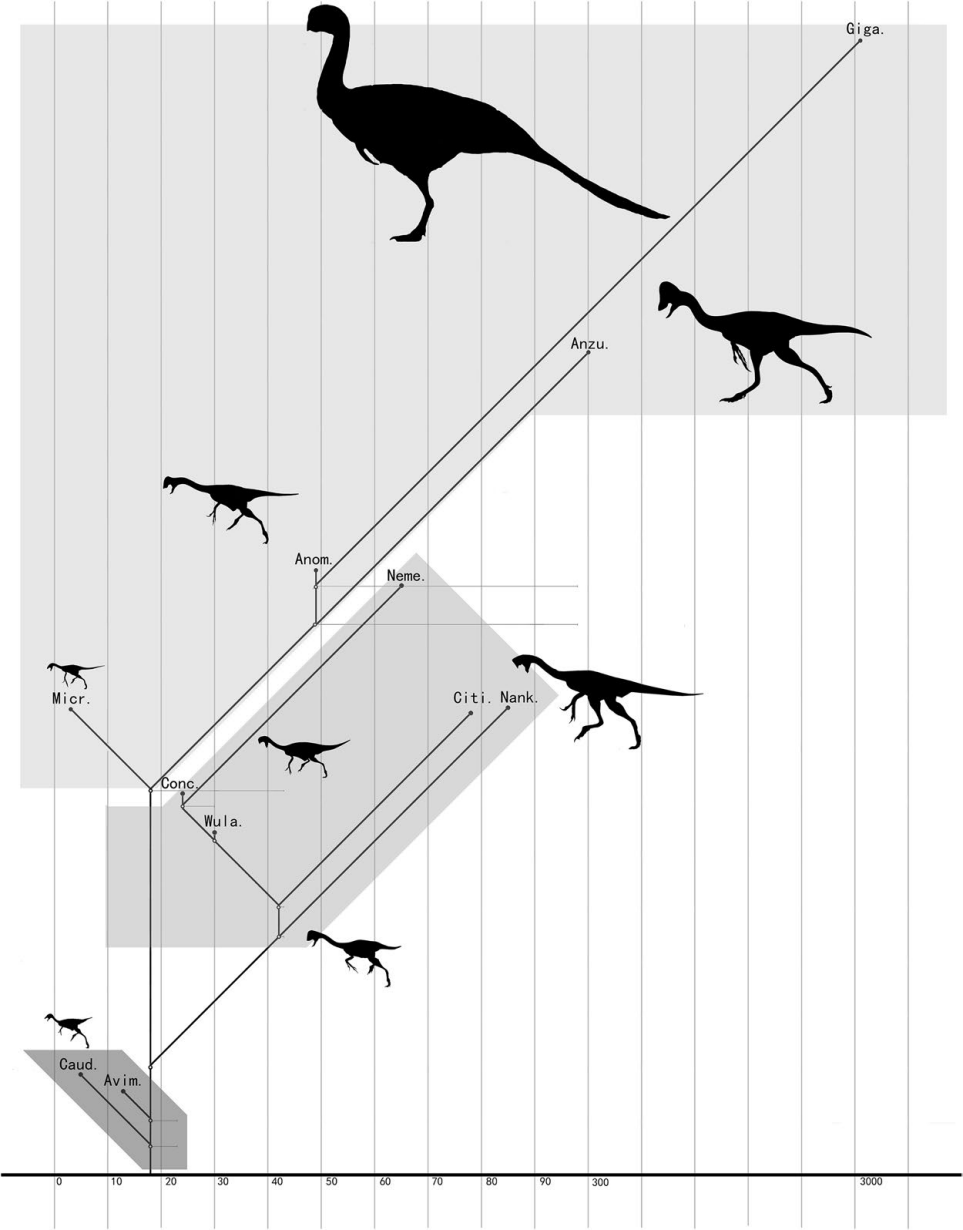
Simplified oviraptorosaurian phylogenetic tree, showing size ranges for basal oviraptorosaurs, the Caenagnathidae, and the Oviraptoridae. Grey boxes represent body mass ranges for three oviraptorosaurian groups: basal oviraptorosaurs, oviraptorids, and caenagnathids. See the electronic supplementary material for estimated body masses of various oviraptorosaurian species. Abbreviations: Caud: *Caudipteryx zoui*; Avim: *Avimimus portentosus*; Conc: *Conchoraptor gracilis*; Micr: *Microvenator celer*; Wula: *Wulatelong gobiensis*; Citi: *Citipati osmolskae*; Nank: *Nankangia jiangxiensis*; Anom: *Anomalipes zhaoi*; Neme: *Nemegtia barsboldi*; Anzu: *Anzu wyliei*; Giga: *Gigantoraptor erlianensis*.

Size evolution has been explored previously in dinosaurs as a whole, and in dinosaurian sub-groups^57–62^. Some studies have revealed oviraptorosaurs and many other coelurosaurian clades, such as Tyrannosauroidea, Ornithomimosauria, Therizinosauroidea, Dromaeosauridae, and Troodontidae, to have been ancestrally relatively small in body size^62–64^. One study further explored size evolution in several herbivorous theropod sub-groups, including oviraptorosaurs, and found no evidence for directional size evolution in these groups^57^. Although we are not attempting to quantitatively assess trends in oviraptorosaur body size evolution in the present paper, the considerable size disparity existing within Oviraptorosauria^8^ prompted us to compile a dataset containing body mass estimates for as many oviraptorosaurian species as possible (see Supplementary Information), using an empirical equation based on femoral length^65^. Although this equation probably produces estimates that are less accurate than those generated by recently developed empirical equations relying on other parameters^66^, we nevertheless consider the equation we have chosen appropriate for our study given that length measurements are readily available and that our central objective is to reveal size differences among oviraptorosaurian taxa rather than necessarily to produce maximally precise absolute size estimates. Our body mass estimates for basal oviraptorosaurs range from 5–18 kg, whereas those for caenagnathids range from 3 to 3234 kg (most species >49 kg) and those for oviraptorids from 11 to 85 kg (most species >30 kg). Although these values are subject to uncertainty, they demonstrate clearly that in general basal oviraptorosaurs are relatively small and derived oviraptorosaurians relatively large, with typical caenagnathids larger than typical oviraptorids (Fig. 5). Furthermore, our data indicate much less size disparity within Oviraptoridae than within Caenagnathidae. For example, the largest known oviraptorid, *Nankangia jiangxiensis*^18^, is 85 kg in body mass, whereas the smallest, *Khaan mckennai*^17^, has a mass of 11 kg. For comparison, the largest known caenagnathid is *Gigantoraptor erlianensis* with a mass of 3234 kg, whereas the smallest is *Microvenator celer*^5^ with a mass of only 3 kg. The largest oviraptorid is thus about 8 times as large as the smallest, whereas for caenagnathids the largest exceeds the mass of the smallest by a factor of about 1078. These differences suggest a wider range of growth strategies and ecological niches in caenagnathids as opposed to oviraptorids, which should be considered further in future studies.

## Electronic supplementary material

Supplementary information
