# Supplementary material for: A new caenagnathid dinosaur from the Upper Cretaceous Wangshi Group of Shandong, China, with comments on size variation among oviraptorosaurs
Source: Sci Rep. 2018 Mar 22;8:5030. doi: 10.1038/s41598-018-23252-2 (PMC5864915; doi:10.1038/s41598-018-23252-2)
Supplement: Supplementary file 1 — Supplementary information [file 41598_2018_23252_MOESM1_ESM.pdf]

Supplementary information to

A new caenagnathid dinosaur from the Upper Cretaceous Wangshi Group of Shandong, China, with comments on size variation among oviraptorosaurs

Yilun Yu[1], Kebai Wang[2], Shuqing Chen[2], Corwin Sullivan[3,4], Shuo Wang[5,6], Peiye Wang[2], Xing Xu[7]

[1]Yuan Pei College, Peking University, Beijing, China

[2]Zhucheng Dinosaur Museum, Zhucheng, Shandong, China

[3]Department of Biological Sciences, University of Alberta, Edmonton, Alberta, T6G 2E9, Canada

[4]Philip J. Currie Dinosaur Museum, Wembley, Alberta, T0H 3S0, Canada

[5]Laboratory of Vertebrate Evolution, College of Life Science, Capital Normal University, Beijing 100048, China.

[6]State Key Laboratory of Palaeobiology and Stratigraphy, Nanjing Institute of Geology and Palaeontology, Chinese Academy of Sciences, Nanjing, 210008, China.

[7]Key Laboratory of Vertebrate Evolution and Human Origins, Institute of Vertebrate Paleontology and Paleoanthropology, Chinese Academy of Sciences, Beijing, China

[*]**Materials & Correspondence.** Requests for materials and correspondence should be addressed to X.X. (xingxu@vip.sina.com)

Supplementary information includes:

1. **Measurements of the *Anomalipes zhaoi* holotype**
2. **Phylogenetic analyses**
3. **Supplementary figures**
4. **Supplementary references**

1. Measurements of the *Anomalipes zhaoi* holotype

	Length	Proximal transverse width	Distal transverse width	Least shaft diameter	Distal anteroposterior thickness	Ascending process of astragalus length
Femur	277+	70.0	?	29.6	?	\
Tibia	325+	?	61.9	34.4	14.5	108.6
Fibula	170+	?	?	?	?	\
Metatarsal III	167	13.6	22.6	9.4	20.3	\
Proximal phalanx	37.4	12.7	15.7	11.3	11.8	\
Ungual	51.4	11.1	3.02	?	?	\

+ indicates incomplete measurement, ? missing data, \ inapplicable.

2. Phylogenetic analyses

We initially carried out an analysis intended to determine the approximate systematic position of *Anomalipes zhaoi* among coelurosaurian theropods, using a recently published dataset from a comprehensive analysis of coelurosaurian phylogeny by Brusatte et al. [1] with *Anomalipes zhaoi* and 13 new characters added in (see below). The complete, modified matrix, which we term the Brusatte et al. Matrix, is given below. Figure S1 shows the strict consensus of 73 most parsimonious trees produced by analysis of the Brusatte et al. Matrix. Our second and third analyses, intended to assess the systematic position of *Anomalipes zhaoi* within the Oviraptorosauria, used recently published datasets from analyses of oviraptorosaurian phylogeny carried out separately by Lamanna et al. (2014)[2] and by Funston and Currie (2016). In each case some characters were recoded, and new characters (see below) and *Anomalipes zhaoi* were added in. The complete, modified versions of these matrices, which we term the Lamanna et al. Matrix and the Funston and Currie Matrix, are also given below. Figures S2 shows the strict consensus of the 860 most parsimonious trees produced by analysis of the Lamanna et al. Matrix.

New characters added to the Brusatte et al. Matrix

854. Femur head, shape: subspherical (0); anteroposteriorly compressed (1).

855. Femur, femoral head, orientation: medial (0); dorsomedial (1)

856. Femur, femoral head, posterior deflection: absent (0); present, femoral head more posteriorly positioned than greater trochanter (1)

857. Femur, greater trochanter, anteroposterior width: less than or sub-equal to anteroposterior width of femoral head (0); greater than anteroposterior width of femoral head (1)

858. Femur, greater trochanter, anteroposterior width: slightly greater than anteroposterior diameter of proximal portion of femoral shaft (0); much greater than anteroposterior diameter of proximal portion of femoral shaft (1)

859. Femur, accessory trochanter: confluent with lesser trochanter (0); separate from lesser trochanter (1)

860. Femur, accessory trochanter: subdued (0); prominent (1)

861. Tibia, distal articular surface, outline: sub-triangular (0); sub-rectangular (1)

862. Metatarsal III, proximal articular surface: rectangular (0); triangular (1); irregular (2)

863. Metatarsal III, anterior flange near proximal end: absent (0); present (1)

864. Metatarsal III, distal articulation, medial hemicondyle, transverse width: sub-equal to transverse width of lateral hemicondyle (0); much less than transverse width of lateral hemicondyle (1)

Complete, modified Brusatte et al. Matrix

Allosaurus_fragilis ?10000?0000000100011001000101200111011001 0??000000
000000100000000000100010000000000001010100100000001001010000000000001000000000
????000000000000000?1000000000000100001001010000000100010000001100000001000000000
000000000000100000000000?0?000?00110000100000010000111000210110000000000200000
0000?0000??0000000?00000??000000000100000[01]0?0????????0000020?00?0000000101000
?0?0010000?000000000000001000010?01000??0000000?000?000002000000000000?00000?
00?00100000000020000?000100??001100000[01]0?0000000010000000011000000000001000
1100000000211210010010001 00?1????0000000000100??10?0000?000000000000011000?100?
000?0?1000000000????000?00010010000101000000000000000001020000000000000000000001
000000000000020101[01]00001000000000000000000000100000010?00010000001020110000000 0
0110000000001000000000000000100100010000000000000000000000000000000000000000000000
000000??0000000000000000000000?00000100000000000000000?00000010?00

Sinraptor_dongi ?10000?000?00010001000000001012000010110010?00?000
000000010000?00000100?10?0000000001010100100000001001010000000000001??????0??
1?10?0?0??00??0??????000000000?010??01001010000001?0?100000110000000100000000 0
0000?000?000?00?000000?0?000?00110000100100010000001000021 01???00000000000000
000?0000??0000000?00000??00000000010000???00?00??00?????2????????????1010???????

????????????????????????????1??????0??000??000000002000000000000000000?00?00??0?
??00020000?0001?0??0011000?????????00010000000000000000010000011000000002102
100000?010?00?100000000000000100??01?0000?00000000000?11000?100?010?0?100?00?0
01000?0010000100100001010?0?0000000000001120000000?00????0000001000?000000201
01??0??1000000???????????000010010?0000?000010201101010?00011000000??1?000000
000000001001000??0???000000000100?00000000000000???????00000000??0000000000000
00000000000?0010?00000?00000000100000000000

Dromaeosaurus_albertensis ?0??001000000000010??0???0?01??01110????1111????101?
1001100??0000001001111100?0000101001???
?????????????????????1??11?1?0????00??????00
?0?00??1?????????1?00??011000100?0???????????????0???0000?00000??0000??0000000?0
0000??00???
?????00??????????????????????????????????????0????0?????????010121?000??0???00?0??
???????0????0??0??0???0??????0?0100?00?00????????????????????????0????00000100000
???????0???????0001001?0?0????00??00?????0?0?10???????????000?1000000010????0000
00000011201000000000?0?00010???
????????????????????0??000?0??????????????0??0?010????????????????????????0200000??
??????????????????????????????????0?1?0??110????????????????000?0??????????00

Deinonychus_antirrhopus ?0010????1???????1??0000?0111000111000111111100?1??
00001110100000?0100111?1000?0100101000?110001100?1121011????00110111121??????1
?1101110010011100000100221110101020220?01111211201?1111000000000100011010010
000000001100000?00010?0010002110000011110110000??00000122000000000?0????000??
??0???????????00000??000000000000?[01]0?0?????????????20000?000?000111000?0?0010
000?0000000000000101?0???00000??0100000?000?000000000000000000000000000?0010
0000000????????0?1????00111?????????0000?????00001?10?0010001?000001010??00?0??
?????00??0?0??0?????00001000000??01?00?0?00000001??????????????????????0?0?10010
00?000?00?????????????????00000000001120100?0000?0?????00000000?00001000100????
?00000?1101010000100??1?0000?????????????????00000?000000000?00?100?00??0001?1
??0?000010?1000000000010?010??00?000000000000000000000000??0????000000000100??
1101?000110???200?10001?0000?????000

Velociraptor_mongoliensis ?0010010010000120112000010111000111000012111?10?010
1000011101000000010011111000101001010000110001100111210111110001101111211110 1
0111111110010011100000100221110111020220201111221201 1111?0000000001110110 1001
000000000110000000011?0010102110000011100110000000000001120000000000000?00000
0?0000??0000000??0000??0000?00000000[01]0?00??000?00000020000?000?000111000?0?
001000???00000000000010100???00000??0100000?00000000000000000000000000000000?
0110000000010[12]21?0001101100011100000?100000000?0100001?10?0010001?0?000101
01000000???0??0?01??00?0????000001000000??01?0000?00000001001?0?0???000??00110
100?0???01000?000?000001001000?00000000000??00?112010000??????00000??????????

????01001000?000001110101?0?0?00?0100000110?01?010000000000000000000000?0001
00000?000?101010?000010010000000010?01002000000000000000000000000??00001
00000000010000?1?10?0011010020001000100000011???000

Balaur_bondoc ???
???1?2??1????0???10?????????0????111[01]
11101001?1?00001???2???0111?202?02?0?1?22122????????????????211000100 1000??00??
1???????????0?00??0??????????????????????1?????2?00??????????????????????????????
??????????00000000?[01]???0????????????20??????0???0111000?0?001000???010?00????
?010?00???21??0?00000020?100??0000??????00???0?000000000?00??0?00000????????010
????????1????????00000???
??
??0100?0?0??0?00?1101010000?00?01000?011
0?01??????????????000?00??00?????00100000?00??1??????0000100100?0000?????0?0?????
??????000000000000000000??000????000000210????????000?10???200?1???????????????0
0

Tsaagan_mangas ?0010010010000120112000010101000 1?1000121111100
010110000100??000000100?1111000101001010000110 0??1??????0??????????????????1????
????11?111??00?
?00??00?0000???????????2110000011?00?0000000?00001????0000000????000000?0000??
0000000?00000???000??????????????????????????20000???0?0?0??10???????????????????
??0????????0121?00???
0??00011?0?????????????00?0100001?10?0010001?0?00010101000?00???0??0?01??00?0???
?000001000000??0??0000?00000001001?000???000??001101?0?0???????????????0001001
000000000000000??00?112010000000????000000???00?00001??????????????00?110??????
??000?000?????????0?10?010?????10???????????
??????02?0000000???????????????????????????????????????000?1?1????110????0???000?0??
??????????

Bambiraptor_feinbergorum ?0010??001000012010[02]00?010111000?1100012111?100
0101?00011?0?0?000?0100?1111000?010010100??1110?1100?1??100?0?1100[01]1011??2?
???0101011011110?001?100000111?211?0101?202202?111022100???111000000000100011
01?00000000000110000000011?0010?021100??11?110??0000?000000122000?00000?0??0
0000??0000??0000000?1?000??000000000100??0??0?0?0?0?0000020000?00000?011?000?
0?0?1000??000000000?00010??????00000??0000000?000?000000000000000000000000?00
?011?100000000121?000110??00011101100?1000000001010000 1?10?0110001?000001000
1000000???0??0??1??00?0????000001000000??01?0000?00000001??1?0?????000??001101
???????????????????0????????????????0?000??00?1120100?????????????0000000?0000100
01011000?0000011101010000 10?00100000110?01?010000000?00000?000000000?0001?00
0??00001?10?0?100010?100?????????????????0?000?????00??00????000000??00001000000
000100?0???10000110100200?10001??01?11??????

Tianyuraptor_ostromi ??01?????????????????0????111?????????????0??1????????
??????????????????????0?0?0?1010??0???0?????????0?0?0?1??01?0112???????1??00?0111
10?001?1100001112?1??0?1102223?21111022?0???1?110??????????00?11????000??0???1?
?00?0??01???1011?0???0??????1?0??????????0????2?000?0????????????????????????????
?????????0??????????0?00?00????00000?0?00?000?0?0????????????????????????????????
????????????????00?1?0????10??????????????0?0????????20?101????????????1?????????
?0000?10??000??1???????????????????????????0?????????????????????????????????????
???0????????
????????????????????0???????????????????????????01?1?1???00??0?0????000??????????
????0?????0?0???00????0????0?0???0???????0???0?????0??0?????????????????0?????0???
0????????????????????????0010????????0?0???????????0?0???????1??????

Sinornithosaurus_millenii
0001??????0??????????00???1110????1000111100?1????1??0????????00?00100??1????010
100??100????????1??1???????0???00?1??1???????01?1?11011110?0?????0000?00?201?01?
112023?2?111022?2?1???1?????????110001?1100?0?00010001??0000???01101111002000
00111100?00?00???000???20000?000?????0??000????0????00000??1000??00?00?0?????0?
0??0???????00?0?[12]0000???0?000100?00?0??010?0??00???????????????????0000??0000
?0000??0?0?00?????000??0?0000?00?0???0111?11000????????01??10000111?1100?100000
0?010100000000?0000000?0?000000001000200???0??0??1??00?0????000000000000??00?0
000?00000001??1????0??000??001101?????????0??????????????????????????001000?0101
12010000???????100?0??????????????????????????0011101????00?000?????????0?01?????
??????????0?0???00000??0??00000?000?1??0?0?????10?????0?00?????0?0???0100??????0?
???00000????001?000?????0??000100?011?????0110????0???000???00????????0

Microraptor_zhaoianus
0??????????????????100???0??010?0?1????0?0100
0000?????001?1??01?2100?01???0110?1212111?01?101111111010??1110000110121111??01
12023?2?1110221201?11111??00?0001110111110?100??000?11?000????1010111100?0?0??
?1?110???0?00??0?00???20??0?000???????????????????????????0?0???????00001000?[01]
0?00?0000?001000200???0?000?01???10?0???10100?000??1?0?000?????????000????????0?
00?0000000000????000?00??000000000??1211111??00?021???011011???1?1?1100?100000
0??????????????????????????????0??
???00???1?????????????
????????00?????????????00001????????01???1?10000?000???0000??0?01??10?????0?0000
0?0???00????0????0?00??0????1????0000???10??0?00???0?010????????0???0?00??000??0?0
00000?000?10?000000010??????????0?1??0?????1???1??00??100???0

Graciliraptor_lujiatunensis ??
?????????????????????020???1???????????????????????????0???11?12????????????????0101
1?1100001?????????????????????????????????????0??0?010?[01]11?1??0??0??00?????????
????0????????????????????00???????????????200???

???0??????10?0???000??0???????????10?000
0??00?000???10???????????????????????????????
?000???
??
??????????0???????????????????????????????1????00?00????????????????????????????00
00????00??????100??0??0???????0?????????00?0?00?????01??????????????????0???00????
????????????0?000?10????????0?0????????????????????????????

Hesperonychus_elizabethae ??
??
?????????????21??010?????????????221211?????????????????????1????????????1?????????
?0??1??
??
???0?????????????????????????
??
??
???01000?0110?01??????????????????????
???????????0???0?????1???000???????
??

Pyroraptor_olympius ???
??0????????1???????????????????????
?001???01??1?????????0???????????
??0??????0???
??010??0?????????
??0????????????????????????????????????
??
??
??????????????????????????????0?0??????????0???????????????????????????????????????
?0???????????????0?????????????????0???
???

Rahonavis_ostromi ???
????????????????????????????0???????????????011121???1?01?011112?12???????????0??1
1??011??????0?01111?10111120?3?2012101?121??21110001000?011101101?0100????0??1?
????0??10001011111??
???????????0011000[01]0???????????????2????????????1100?????????????????????????010
100???????????????000000?000000000100000000?00000000?001??01???0????????0?1?11?
????1????????00??
??
??????????????????????????????????????100?1??0?0??0011??????0?????0?100000????01?
?10???0?0????00?0???00???????????00??0?00?1????0000??0?0???????0???010???????????

000?????????0000000?000?10??00?0001????????00?0?11?0??0???????????????????

Buitreraptor_gonzalezorum ?0010???????????????00001011?00??????????100?10??01?
0?????????00??001??????????0210??100?0010111100111?110???100011012[012]121??????
?01101111010?1?1??0??????11?101??12[02]23??01?1?1?120???11?0??0??1?011?0?1110000
?????0??1????????1101000110011000???0??????0000??0?00?10200?00?0???????????0????
???0?000101???0?????00000000000[01]0??????????01000100?0?0?00??0?10000?0?001100
??00000100000001?100???????????????000????00???0?00??????????0?00?000?0010?00???
???????????1????0010??1100?0000???00?0?????????1?11?00?0???010?0101?0??0????00000
??1100????????0000???0?0??0??0??????????????100?????000??00?0?????????????????????
0????????????????0?000???0?1?2?????????????????000???????0001001000??0??0000011101
0100???0?001??000???????01000???00??0??????????0?0?000??????????00????0?1000100?0
???????01000?????0?00010000000000????000000??0??01000?0??001???????10????11100?
00010001??00????????0?

Neuquenraptor+Unenlagia ???
??11211111111???1????1?????????????0??10
01????????????01111?10111120220201111?121202011?10000??0?010??111100000????0??11?
???0??110110?110???????????????????????0?????220????????????????????????????????????
????????0?00000?0??????????????????1?????????????1100?0?0?0011000?00000????????????
0????????????????00000?00000000000000000000?00000000?00??????????????????????????
???
???
???????????????????????????????????11011?0?????0011???01???????00100000110?01?010
000000?00?0100???00??????????000?00?00?1????10????01???????00???010??????????????0
0???0????000000??000010??0?000010?????????0??111001000?????????????????0

Austroraptor ?0000????????????????????2?1?0????????12?0001????????
?????????????0?011????????????210??10?????0011?001????11??????????????????????????????
??00?????????0??????????????????????????????????1??????0?0?0?0??[12]1???????????0????
0??????????????????11?00??01??00??????????????1[12]20??0?0????????????????????????????
??????????0??00?00????????????????????????????????????0?0?001100???00??0000000???
???
???????????00?0???????????0?10100?0000010??1????????1??????????????????????????????0
??01?01?????????????????????0?10?00?????????????00000?000?00??????????????????0??0010
000??????????????????????000???????0001001101???????????????101????????????????????????
????????000????????00?00??????????????0?????0?0?????????????????????????????0?0001000?
????0????????0000????????00???????0?????01?1????1?????1?0??0001?????????????0

Shanag_ashile ?????????????????????1??1010110?????????????????????????
?????????00??011???????????01001011??
???0????0???

?????????????21100001???00??????????????????00??0?????????????????????????????????
??
??
??????????????1?10???00?0?000000??
???0??00??00??????????
?????????10???
??????0?0????????????????????0????????????????????????????00????????????????????????
?????????????????????1?????????????????00???????????????

Mahakala_omnogovae ???????????????101??????????????????????1?00???????
0?0011??????????????????????02????1???00??0?11?011?211??1?10001101221 0??????????
?????00??1110000?100?211101?2??????????????????0111100????0?011001101?00000??00?
?11?0?????0000?????00????????0?????????????0????1[01]2?0??????0?????????????????0??1
?00???????????0000010000[01]0????????????????????????????10??[01]0???0?0000???0???
??????????100????0??0???000000??0??001??0???000000000?00000000?001??00?000????1
???0??????011?0????????000000???????????????????0???0??????????????????????????????
????????????????????????????????????0?????000?00????????0???????????????00010?????
????????????????0????????????????????0000?????0?01?000???????000??????????00????00
100000???????0100000?0?000?100?0?000?0????0?0?0???0????1?10???00???????????0????
010?20????0?0??0???????000000000??0??010000000010?0????1?000???10????01????0?0
0011???000

Atrociraptor_marshalli ???????????????????[02]00?0??1010??????????????????????
??????????00??010????????0?0000101001??
??0????0??
??????????????21?00??0??111?????????1????????????0??????????????????????????????0???
?0??
??
?????????00001?1????1???0?0?000?01??
???0?????0?00????????
????????????0???
????????0?0?????????????0??????0???????????????????????????000????????????????????
???????????????????0??1??????????????????00???????????????

Utahraptor ???????????????????[02]00?0??????????0?001???????????
???????????????????????????0????0101??1????0?1100?1??1????????0??011?????????????1
01?1????????????0?????????0?01????????????11?????0111?00??0?001000100???10????????
11??0???????0?????0????????????1???0???????0???10???0???0???????????????????????????
?????????????00000??????????????????20000?000000?????????????????????????????????????
?????????????????00???????0??000000000000?0000?????0?10???????????????????????????
????????????00?????000001???????????????????????????1???0??00010?0??????????????????
??

??
????01?????????0?0?????????????0?????0???????????????00????????????0??????????????
?????????????????0?0???????????????????1???????????????????????????

Adasaurus_mongoliensis ?0010?????0????2??????????????0?????0?1??11?1?0?101?
?001??0?????0????0?1???0?????????????01100?11?0111?10?111[01]100??011?1?1??????1?
111111??????????????1022111010102?2202?1?11221001?111000000?000?11010010010??0
0??00110?0?0?0001??00100?????????11????????00?0????1??????????0?????????00??00???
000?0????????????000?0010000[01]0???????????????200?0???00??01110?????????????????
????????????????????????????00?000000000000000000000000000?002??11?00???00???21?
000??0??0001?1?????????0???0??0????????????????????????????????0?????????1??00????
??0000010??000??0??00?0?0?00????????0??????????0???????????????????????0?????????
?????????0???????11201???10
0000??0?01???????????????0?0???00?0????????000?00????10??????????????????????????00
??????0??00???????????000000??000010000??00010??????????01???????????????????????
????

Achillobator_giganticus ????????????????????????01?1???????????????????????????
??????????????????????0000101??????0?01100?11210???????0??011?11??????????101???
?????????00??10220??11010102102?011011?001?21110?0????00?000?101????0???????1???
0????001??00100021100??0??100????????0???
??????????0000????????????????????20000?0?0?0?0??10????????????????????????????????
??????????????00???0?00000??000???????0?0000????????0???????????????0??????????0??
?????????00???????????00?001???0?0?000??1???
??
?????????????????10???????????????0100?????????????????????????100000??0101?010000
0?1000??????????0??????????0??01?????1??0??0???????????????01??0?????????0??00??????
???0000000???0000100???????1????????????????1?1?????0?00??????????????

Saurornitholestes_langsto ??111?????????????
11????0???????????????????100101?00?11000110011121011011100?1011?1?1?????????????11
1?????????000111221?1?1?1?2?0??????????????????11??0????00??001101??00???00?0?1??0
0?0000010?001000????????1110011??????0????1220???????0????????????????????0000???
????????0?????????????????????????????????0???00??1??????????????????????????????????
??????????????0???0???00??????????0??????00111???
?????00?00????????????00?01??0?0?0?000?00??
??????????????????????????000??00?1???0?000????????????????????????????????0??0000000
????????????????????0????????????????1?0???
??????????????????0??????0????????????????0????????????????1??01?????????????0????????
?00?????????????????????????????11???????????????????0????????????????

Saurornithoides_mongolien ?00??1?1??1101???0?110001?1000????????2??????????????

???1?010?100?0010??1????0001110101?????????1???0??1?????100??1??????????????????
??????????????????????????02012020?010??10[01]1?11110???????????0???10?01??????0?
?1?0???????????010??11000??0?1?01?0?????0?000??????0??0000????0?1000?0??0???????
???0000???0????00100?0??
??????????????????00????????0?0????00000000000000?20?0210000000000201001?10??
?1110?1????????????00?0100000??2??11?0?0?1?0100010100??00???0??0?????0??0???????
??????00?????????????????????????????????????0???????1????????000???????????????
0?????????????????0???????00000??????????????0????????0?0??????????????????????????
???1?010???????????00????00000????????00??00????0?0???00???????????1??????0??000?
0?????0???????????????????0?00???????0?101?0????????1???0??????0?0?0001011?????0

Zanabazar_junior ?00101?12?110100?001?000??100000????2022000?21??0?
?11100??????100?001???1????000111010100??????????????????1?1000?1020?1??????????
??????????????????????????????2?????????????????????????????011????2???????????00??
00???11????????0??110000000100000????1??00???????00?000?????001??0?00??????????
??0000??0?00?????????0[01]0???
?????????????????????????????????????00010000??000??2????1??0???00000201001?11???
1110?1????????????00??100000??2??11?0?0?1?0100010100?000???0??0?????0??0????????
?10??000??00?0??????????????????????000?00011010?????????????????000?00000????00
00???000?001???????????0????00000???????????????????0??????????????????????????????
????0??????????????0?0????????000?????????????0?????00???0?????????????????01?00?00
00?????????????????????????????????0?00????100100???????????1????000?0??????????0?

Xixiasaurus ???????????????????01000101110??????2?22????????????
????????0?00??001????????000201??01?0???
???????????00?1??0???00???????
??????????????1?000000???01?0000????00???????000000???00????????????????????????0?
???
0??????0???0?0?????????????????????????????
????00????110100??2?01100?0?1?01000101000000???0??0??????????????????????????????
???????????????????????000??0???010?000???
?????????????00000??
?????????????000??????0??????0????0?0??????????????0????????????0100???????????????
0?????????????????????????1?010??????1??????????000??????????????

Byronosaurus_jaffei ?????101???101?1100110001011?0??????20220??????????1
?100??????0000001??11????000211??01?0?0???????010121????????0??02????????????????
???1????0?0??0???????21??????????0????0
????1???????????11000000?1?00????0??0?000??0???000000????????????0??????????????0
?0????0????0010??
?????????????????????????????000?????????????????????????????000200011??0????????1??
??????????00??100000??2?011?0?0?1?0100010100??00???0??0?????0??0?????????????00?

??????????????????????????0???????????0?????????????????00?000000????????0??00??0
0?????????0???????0?0000??????????1??
?????????????????000?????????????0????000???????????????????????00?0100?????????
??????????????????00?????????1?0?0?1????1?????????000?0????????????

Sinornithoides_youngi ?0??01?????????????1?000??1??0????00???2???????1?????
?????????00??0010???????0001110?01??????11??001???????????0011?102121011???1?00?
1?101?0???100000????21??01??02??30???11?0??001??1110?????0???1?000110001?0??0?0
?1??00?0???0???0010?0??00000????0??0??0???00???0?2000000?0???????????????????00?
????1?00??000??????????[01]0?0????????0??0?20??0?00000?0??1000???0?1000???00?0???
??????0??010?0000???000000??00??0??000????000000000000000020?02100000000???????
?101??????1?010100??1??000?????????????????????????????????????0???????????????????
???000??0??????????????????????????????????????
??????????????????????????????0?0?00?????????0???????????????00?110101?000?000????
???????????1??????0?????000???00?0???0???0?00??0??1????0?????100????0?0????????0??
?000?000???00??10?000?????0??00???00000000010????0?1?0?0101????0????0???????????
????

Mei_long ?0????????0????????11010?????0?11?002012100001?10
10??100??????00010010??1?0000?021???01?0011011100011?2110?01???0110222111?1????
?111?11100?0?1?1000001?012111?1??1200302?1110221?[01]?0111100?0101001000??1100
11?0?0000???00000??000100?1??0?1?000000?001?0?0000?000001020000?000????????00
?0??0??00??0101????????0?0000010000[01]0?0????????0100020?00?00000?0111000?0?00
00000?0000000?000?01?1?0???00000????00000?00000010000??00000000000?000021?011
00000??0???20???1010??101100?1100?10?0000?110??0??0??????????0?1?01????0?????00?
??????????????0?????????????00?????????????????1???0??????0????0????0??????????????
??????????????????????0?000?????????????0???????0?0000?????????0??????????????????
??1?1?0????????????????????????????0??????00???00?00?0??????0????0????1?0?000?1001
??00?0001?0?010???0???0?0000000000?000????00???0???00?000000100?????????01?????
?????0?0???0??????????

Jinfengopteryx_elegans ?0?????????????????1?01???2000????????1??00?0????1???1
00??????00?00??0??1??0?000?1???0???1??????????????????????02?????2?200???????11011
10??????100000?????1???????????????????2??0??????????0?????????????????0?0??00????0
0??????????????01?000001???0??0?0??????0????200?0?000???????????????????????????0?
?????0?000???0?0??0?0??????????????00?0???0?0?01???0??????????????????????????????
??10?0000???????????0??11?01??????
???????000?110?????0?01??1????0?????????0?????00????????????????????????????0?????
?????????????????????????0????0????0????????????????????????????????????0??00???????
??????????????????0??????????????0????????0????1??0????00????0?????????????????????
0???????????????0?0??00??????00????1?0???0????????0????????????????000?????????????
?0?0???????00?????????001?0?????????????????????000??????????????

Anchiornis_huxleyi
000100?????????????11010??11100?????0?2?000??1???11???????????000??01??1?????0002
0???01??0?101???00?0??010?01???01101220??01?????01111110000??100000010?21?1001
1020?202?1?1122121???1?1?000??1?01?000121100010?1000?1??00?0???????0?11?011000
001???0??00?????000???030000?000??????????0??????0????0??????0??????00000?0000[01
]0?0????????0000020000?0?00??0?10000?????10?0???0????????????????10?01000??0?00?
00??00?00000????????????00000?0?00??0100000000????????00??1110?1?1?1100010??000
?110??0??0?01?010?0?0?1??101?00??0??00???????????????0?????????1???000??0???2????
??????1?????????00????0????0??????????????????????????????????????0?000??01?????????0
???????0?0000?????????0???000??????000??????1???00??0000100000??0?01?????????0???
??0?0???00?00?0??100?00??00???11?0?0000???????0???????????0????1000????0????0??0?0
000000???00?0???????00100???0???0?01??????????000???00?????????0

Xiaotingia ?00??????????????????1?1???111000????0?2???????????11?
???????????001001??1???00??020???01??0??0???1?0?????10?01?????1??????10??????01?0
1111?10???1000001011?1110?1102122?????102?12[01]??????????10????????1?0100010100
00?1??0?10??00???0010?011000??1???00??0?????000???22000??000????????????????????
?0000?????????0??000?10000???0?????????010002?????????????11?000?0?00???0???0?0?0??
??0?????????????0100??1?00000???0?0000????????????????????0??0?02????10000????????00
??1??????1?1100?10??000?110??0??0?01??1????0?????0???0?????0????????????????0?????
????1???00?????????????????????????????0????0????0????????????????????????????????????
??0??00???1?????????0????????????00??????????0???????????????0011??????0???000010?00
0????????????????0????????0???00000?0??100?00??00?1??1?0????????0????0????????????0????
?000?????0???0??000?000?????0????????????0?100???0?????0101????0???000???00?11?????
0

Aurornis ?0010???????????????0101???11100???000?1?0???????????
?????????????00?001?????????0?020????1??0?????????0?????????0????0??0?221?00??????01?0
1111??0???10000??001?011????02122020??10???????????????????????0??100000?000000?
1??0??0??00????111?011?0000????0???000???0000???20000?000???????????????????????
???0??????000??0???000?0??????????00???2??0?????????0?0?010????????0???????????????
?????10?0??00??0?00?00?0000000????????????????0???0??????1??010000????????0???1?1
?1??1??1000100?000?110??0??0?01??10???0?1??101?001???000???????????????0????????
?10??000??0??02????????00????????????00????0???
0???0???1?????????0??????????00????????????????????????0???01???1???00??0000100000?
??????????????0????????0???00??0?0??100?00??00????1?0???0?????????0????????????0?????0
0??????0??????0?000000???????????????00100????????????0?????????00?????????1??????

Eosinopteryx
00010??????????????1?01??????000????0?1?0?????0??1??????????????00?001?????????0002
0????1??0?????????0??????????????0??0?231?10???????1101111??0???100000?001?1?1??1?0
2002?20??10???????????????????????00?1??0001000000?1??0??0??00???011?0???00?????

?00?0?00???0000???20000?0000?????????????????????????0??????0000?0?10000?0?0????
????00???2??0????0???0?1?010????????0???????????????????????0??00??0?00?00?000?00
0?????????????????0?????00??01???000??????????0???11??1??1??1??0??0?000?11???0??0??
?????????????????0?????00???????????????0?????????10??0????????????????????????????
??0????0????????????????????????????????????0???0???1?????????0???????????0????
??????????000??????0??0011??1???0???000?100000???????????????0?????0?0???00??0?0?
?100??0??0??1????0?0?0????????0??????????0????100??????0??????0000000???????????
???00100?????????0?01????0????0???????????????

Troodon_formosus ???1?1112?1101000001???0?011?0??????20220000210?0
??01100????0?10??001??????????0111010100???1111100101211111?1000?1020??11???????
??????1?010?????000010??????????0?0?2?20?11001?0??01111000???00010000021??01????
000??1?0??010???1??0??0?1?00000?0?0???????00??????????????????????001??0?000?????
0??1?1?????????00??
??0?????00000201001??0???
1110?1?????????0000??
?????????????????????????0?????0000??00110?????????????????????0000001000??000000?
?0000000?????????????????????00???????0?0??00??????????????????????0?????????????????
????????????????00000?000??0????????00??0???0??0????00??01??0000?0?1??01?01?00?0?
???????????0?0??????????????????0?000?10?0?10?0?0?0????????????0??0??????????00

Sinovenator_changii ?0???0002?000011110010101?1110?011?02??2??0011???1?
10100????0?000?001??????0000011110?1?????11?10100012110?001000110222?1?????????
110111???????100?0???0?211?010112[02]0302?1110221201?11110000??10011000111??110
?000000???0000110001000110011000??1??011??0000?00000??0??00000000000?000000?0
0?0??0000010100[12]00??00000?0010000??????????????????20000??00?0101?10??????????
????????????????????????????????????00?100000010000000000000000?0?00?020?0110?0000
0000020?010?10??1011000???????000000?????0000?02?011??00?1?0100010?????0???????
???0????????0?00????1???????????????????
0????????0???00000??00???0?????????????????????00?????????????00????0??????00111???
??????????0000?0??0?01???????1??0????0?0?0???0?0????????????0?0?1?1??0?????100?????
???0????0??01?01?0?0000?00??????0?00??0???0000?0000000001?1???0?????0??1??1?????
000?0?????????100

EK_troodontid_IGM_100/44 ?????0012???
0???????????????1??11???????????1??????0???
???????1?00000??01?1?001???00??????0?0
??1?????????0??1?1???????
??
??0?????????
000???
??????????0???

??
??0??????????1????0????????????0000????????
????????????0?1??0

IGM_100/1126 ?0????002?0000?11[12]01?010??2000?011?0201210001
10?01010100??0??000000000?11?000000211??00?0?????????????????0????01102????????
???????????????0??1000001???211?010?020020200010131012??111000????0??2?00121000
1?00000011?00?0?020000?00100?1?00000111?01?0?00?000003??????000000?????00??00?
00?0??0??0000?00?00???0?0?????????0??
????????????0?00???0000???0100000?00??0???0?00?00?????00?1?000?20?02????00000010
20?111??0??101?001???????0?000???
??
??
??
??

IGM_100/1323 ?00???????0???11???1?000??20000?1??020121?00?1??
0?0??????00?0?000?0????1??00000021???0000??????????????????????011??????????????
?????????????????????00??1??????020?20200[12]102?1011???????00???0????0??2???01??0
0??0????0?0???00?0?00100?1?000000?1?01?0?00?00?003??????0000000????00?000?00?0?
????0000?0?000??0?00???
?????????????????????????00?????00?00?0?00?????????1000??2????????????????????????
????01??
??
??
??
??

Archaeopteryx_lithographi
100?0000??000??112010010??1110?011000012100?10?0000??100111?0?000001000002?00
000200??00100?1?1???00?0??1?0?0???0021012311000????1011111111000?1100000001121
11010?120030201210 22?2?2?111100000?0001000000030?000000001100000??00?000110
011100001001?01000000???0000?030000?0000000?00110??00?0??000?00??000???00?000
000000[01]000?0????????00000?0000?00000001000000?0011000?000??000?0000100000?
?00000?000000000000?0?00000000?00?0?[01]1000000?00???0000010000 1020?000010111
01?0101100?10000000110?00000?01?01?0000?0?0100?10100?000???0000?01??00?0????00
???10??000??????????000000?0?0????????000??0011000?????0??????????00000011??????
00000?000000000??00???0???0???0?0100???????000?0000????????0?0011101?10000?000?
100000??0?01?01??????0???00?0??00?00?00?100000?000?1?1000?0?0010010??000000?
0?010020?1000?000000??0?000000?0000?000?????0?000100001001???0111???00?10001
0????????????

Confuciusornis_sanctus
10010??????????????1?000?00??0001???0??2??0??0??00??01???????000010000?10?0001?1
?????????0??????????1021?0?2???0?2????4??111?11010??13111000?11110000001121?1?1?
?12000?2?111023?2??12??10??11??120211010030?0000000001102000??00?0101??11?1000
11001??????0?????000?123001[01]00120?????????10??00??011200??01[12]0111110000001
100001001000120000?00?0000?0??0??010000000?000211??0010000000010102101011[01]
?10?0000001010000000001010100100011000011000?0000000[01]000???2000001101110?0
0??1100?10?0000?110?00000??1?00?0000?0?01?0?10100?00??????????1??00?0????000000
0??000??01?02???00100000?0????????000??00???0????????????????????????????????????0
0010???0?0??00??10?????????????0???????0001?000???0????0?00111?0?1?000?100??????0
??0??1?????????0????00?0???00?00?0??100000??0??1??0?????0?????0??00???????010????
???0?????0?0000?0?0??????0??0??????0?0000100?0?0?????01???????????1??????????????

Jeholornis_prima
10????0????????1???0???0?????001??????????????????0???????????0000?1???00?00?1?1??
???????0????????0????1?0?11???02111031??01?0???011031110001??1000001011?1??0??01
?0???2???1?221?10???????01????010110100300000000??0??1000??00?010????1???000?1
?????0???0??0??0??12300?10010??????????????????0????00?00000??00?000000100000?0?
?001???0000?00000?0?100012000100 0?0010000?000?0000?000010000101 10000?0000000
00000?00000?010000000001000001000?00000001100???????011?1110???1?1100?10?000
0?110?00000??????????0?0???????0??????????????????????0??????????0??00?????????????
???????????????000??00??0??00???0???????????
???????????????????0?0??0000???????0?0011101?1?0???000???0000??0?01?????????0????
00?0???00?0??0??100000?00??1?10???0?00???????00???????010???????0?????0???0??0?00
0?00???000?????0?0000100???0?????0??????????1?1?1?????????????

Jixiangornis_orientalis
100??????????????????0010?????001????0?0??00000??????0100??????000001????0?0??1??0
????????00?0???1?00????1??20???02??1031??11?1?0001103111000110100000111121 01010
212000020101?221?10?2?11?001??1?01[12]1101003000?00?000?0??1000??00?0100??111?
001?1?????????00?000000?12300?00010??????????????????????0?????000??00?0000?11?0
?10?11?0?????0000000000?0010011210010?0?0000000?000???0??0????0??010?00100??00
00?001000000?00??0000??00000100000?00?0000?001100????????00??11????01?1100?10?
0000?11??00000??????????0?0???????0??????????????????????0??????????0??0??????????
?????????????????????????0???00010???0????????
??????????????0?????????????????????????????????0?1?00??00???????????????????????0????
?0?0???00?0??0??100?00??0?????0??????????????00?????????0?????????????????0??0?0??
??????????????????00100???0?????0?????????????1???????????????

Yanornis_martini ?00?????????????????1?010?????0?11???0??2??0??????00??
?????????0000011???0????000?1???00??0????1???0????????4?1???????04??????110?01103
1110?011?130010??????????????????????????23?010?2??10??1??0?0121[23]0000300000??00

01???00?0???????0???21?1?0??????????????????0?????300100012??????????10???????1???
??????????????0?0011?00???0210?[12]??10100001010?1?11?112100111??0101?0101?1000
0?0?????021[01]101311[01]1100[01]100?0?1?0??0?00??????0??00?1110[01]?1?00??1?0??0
1100????????01??1????????1100?11??001?11??????0??????????0?0???????0?????0???????
????????0??????????0??0???
????????????????0???0???0??????????????????????0???????0?0??0?0?????????00111?0?1?
00??100???0000??0?01?????????0?????0?0???00?0??0??100000??00?1??0??????????????00
?????????0?????000?????0????????000?00??????????????00100?????????0??1?????????0?
1?????????????

Apsaravis_ukhaana ??????????????????????0??????????????????????????????
?????????0002?1?0??0????????????????1??11??10200??1???5?100021??24??????1?0101103
11000011113003??000?1???1?201000?2?021?23?03?12?010?????1??12130100?00000??20?
??1?20?0?????0?00??21????????????????????????????300?10????????????????????0?1[01]
?????1?0????????20010?[01]001??21??1????????00000?0?1000121011111?01011010[12]01
11110000101021110?[23]120100?11??20?1[01]0010?11?110?1012112111111?0000?1?0??01
101????????011??????0?1????????0001???
??
???0?????????01???0?????0??0?001110001000
1?1?0?1?0000????01?010??????????0000??000????????????0??0??1????????00?00100?????
0??????0???????0????0000000????00000????000????0?000?10?????????0?0?11????0???????
?????????????

Yixianornis
100?????2?021??1?????0?01????0?1?1?0????????????????0100??????000?010???0?00?0012
0???10?01???1???0000??1?0?40??0???0024???11?1100011031110?0010130000100021?101
?212003020001023?23?0211?0??1??????2130100100000??000?0??1000?20??0?000001?????
???????0??0??0???0??0?1230010001???????01??1????????11[01]?????1?0?????10?2001100
0?11021??[12]??10100001010?11110112100111100?0110???01?000???0?01021[01]10?3120
1100[01]100?00100?00?002?101??????11110[01]?1?00??1?0?001100??????00011?11?01???
?0100?10??000??1??0?????
?????????????????????????????0???0
??????????????????????0??????????????00?0????????00111?0???000?100?1??000????01????
?????0?????0?0???00????0??100?00??0??1?????????0???0????00?????0???0???????0?????0?
??0????000?00????00??????0??00100?????????0?11????0??1?????????????????

Sapeornis ?001???????????????0?010??11000????00??21?0?00????0
??????0????0000010??10?00000020??????00????1???000????0?211??0210004??100?????21
10111101001110000010112 1??00?212000?201[01]00221210????1??01??1001210?10030000
000000????2000??0??0100??0111?000???1???00??0???0000??23000000000??????????????
???????????0?00???000000001000010 0????????0000020000?00000?010001000?0?01010?0
010000?0???010?1[01]10?11100??0[01]00?0000?0000?00?000??00?00??000001?00??0?000

01000????????001?1?1011?1?11000100000??1?000000??????????0?0???????010??00?????
????01??00?0?????????00??0??????????0?????000??0????????000??00??????????????????
????????????????????00000???0?????0???0???????0?0100???????000??000?0??????0?001
11?1?1?000?000???0000??0?01?0???????0?????0?0???00?0??0??100000?000?1?10?0?0?0??
0?1???000000?0???0????1??0???0?0??????0?000?000??000?????000000100?0?0?1???00?1?
????????1?1?????????????

Neuquenornis_volans ???????12????????20????????????????????????00?????00
???00?????????????????????1?11011?10311
10?01111000????????????????????????????????????010???????????[01]010030??0????0????
??0?00???????????1???????????????????0???????0200????????????????????????????????
???????????0110???????21?????10110??0100?001?001?0??[12]??1???01?0???0?????[01]???
??????2???20?1??01????????????????10?1????????1?0???1011???????0?000???200000110?
?????????????????0??0???
??????????????????????????????????00???0??????????????????????000??0100??????????
???0111?????0??????????????????
????????????????0?0???00???????????00??0??1???0??????????????????????????2?????????
??????0?????????????????????????0010?0????????0????????????????????????????

Patagopteryx_deferrariisi ?0??0010???????????
??2???????????????????????????????????????0?
1???????????211101?????????????????????2?010001??100121301001?000???????1????????
00?0?????1??????????????????????0?????0??????????????00?1??????00??111110000??????
???010000000????????0????????000????0101?1210[01]00???[01]?10?0???01000001[01]000
1?[12]00???30???????00?2000000?010120????0?000[12]11001100000?[01]?0??0?100?1?20
0??01?0???0?001????????00???0?1???
???????????????????????????????????0??????0?0??0?110????????????????????0??????????
????????????????0020000??00??????????0?0?00??0001?00000????????00111?00100???????
1?0000???????0100??0?0???0?000???00????0??????00??0??1??????0?10???????????00???0
10???????00000000??0?????000000???00?1???00000010??????1???0????0???0?1???10????
????????

Cathayornis ?0??????????????????1?010?????0?1??????????0?????????
??????????00?????????????00001???????0????????[02]????????3?????2????4???0??11?02?1
031110?011?1300?0?01111?0?00?12000?201?1022?23?????????1???0??[12]1[01]1?0?3000?
0???00????0??1??0??0100???111?????????0??0???????0????2300[01]0?012???????????????
????????????0??????0????0111??0010?20??1??10110?101????010?01200010010110[01]?01
020111110?[01]?1?????210120?11?01?0101011?0?000001???1?0?0?1[01]1?0?????????????0?
?01110????????01????????01??????????0???110?00000??????????0?0?0100?10100?000????
???????????0??????????0??00??????????????????????????????000??00?0?0????????????????
????????????????????0??00??00?????????????????0?0100???????0????????????????00111?
0???0?????????0000???????????????0?????0?0???00?0??0??????00??00?1?1??0???????????

????????????0????100??????0???0?????00?00???????????????00100?????????0???????????
000??????????????

Concornis ???
???00???????????????????0??11?02?10311
10?0?1???0030????????????120?102??11?23??3?0???1???1???0?1[02]11??0?3000?0???0???
?????????????001??1????????????????????????????2300??????????????????????????????????
???????????0111????10?20??1?0101100101?0?0010001?0??[01]001?110??0102?1???10???1
[01]1?[12]??????????????[01]????????????00?????0?100??1?00??1?[01]1??0??????110?????
???????????????1100110??01???
???
???0??????????00111?0?1?0????????????
????????????????0?????0?0???00???????100?00??0??1????????????????????????????0?????
????????0????????0?????????????????0??0010??????????0?????????????????????????????

Gobipteryx ?0?????????????????1?01010?0?0??????????????????????
???????11?000?0??????????1?1???2??????
??11?????????????????????????
???????????1????0??????????????????0????????1100100?00110?10?????00?????0???0??0??
?1?????????????10??????????11??10000?0?100012000[12]001?1?0110???0????????????1?1
21??[012]0?????1?0??1????????000?0???10?001111000?1?11?0??????????????????????????
?????????????????????00000??????????0?1?01????0???0?0?????????????????????????????
???0????????????????0??????????????????0?010
00?000??00???000???
????????????????????????0??0??????????
??????????????????????????????????0???0??????????????????0????????????????

Vorona ??
???
???12?0100111010011121110???00??????????????
??????0?????2???
???
?????????????????????????1010111100001?000000??
00???
???
???1???????000000
00?00???0?10001?????????????????????????????
???????110?00000010??????????0????0?????1???1?????????????

Songlingornis ?????????????????????0?0???????????????????????????????
????????00?????????????000?0????0???11??[01]???3?
???0????0?????0?????00?

???????????????????????????0???????0?????????0?01?????????????????????????????????
?0????????????????21??[12]?000100?010???1?11011????????????????????????????????????
??0??0?????????????1??1????????????
?????????????????0???
??0??00??00??????
???????????0?0?0?????????????????????????????1?1?????0???????????????????????????????
?????????????????????????????1?????????????????????????????????00??????????????????
??????????????????0?1????????????????????????0???????????????

Pengornis_houi ?0010??????????????10010??0?00??????0?12??0??????0?
????0??????000?001????????0?0?00??00???01??????20???0???2???????????4????1??????2?10
31111?0??????03??001?1???????????????????2??2???????????1??????211???0?000??0??000??
?0??0??00???0???11?1?000??????????00????0000123000 0?000??????????????????????????
???0?????0???0011??00100????????01[01]00?00??????0??1??012111[01]0?011010001[01][
01]1?????1??1???10[12][12]0??1??1?11??????0????00?0????0??0[01]?1?00??1?11??0?0??0
?100????????01?????00?01?1001011???1??110?00000????0??0??0?0?0100??0?????00?????
??????????0??????????0??000??????2??????????????????????0?0??0??????????????????????
???????????????????0?0?0???0?0??00???0???????0?0100??0?0??0??1?0?????????????00111?
0?1000????0???0000???????????????0?????0?0???00?0??0??????00??00?1??0?0???0????1?
??????????????0????1?00??????????0??????0?0?????00?1???????00100???????????1?1???????
?000???????????????

Hesperornis ?0??????????????????00010100?00??????00021?001????00
???00????1?000?001???1?0001?021???00??10101110?2100??11?50100?210?24??11??10??0
0103111??????????3??0202100?1??01000???021023?03?12???0001??11?1213110010??0?00
??01?1?0000?000???00??2??1?1111????0???????10000??10?1?10?1121200110?1010??1110
011100??00?0???1010121011000012?0?000100100?0???10?0???00011??0??????????0???0??
??0???????????????????????????20?11000011121102111100211111221220 02?0?????00?????
?0001?0???0?000?0100?11???1??01??00000??????????0?1?0100??0?????00????????0???0?
?0???????????0??00?????????????????00??0?????????0?0??0????0??????????????????????????
??????????0?000???0?????????????????????000???????0?0??000???????0???01?????????????
201??000????01??????????0????00?0???0??00?0??????00?????1??0?0?0?0???0?????????????
??0?????000??????????0?????0000?????00??????????001?0??????0???1??????????000???????
???????

Baptornis ??
??2?????????5?????????24??????????????????
???212?????????????????????????
??????????2????????????????????????????????????[12]????????????????????11001??00??1?????
?????121011000012????012?0?????01?10?0???000????0??????????0???0??????????01?[01]1
[01]????????????????20?11000011121102111100211111?21020?2??????????????????????????
???

??
??????????????????????0???????0?0??000???????0???01???????0?????201??000????01???
??????0????0000???0????????????00?????1??????0?0???0???????????????0???????0???????
??0?????0000?????00?????????0210???????0???????????????????????????????

Ichthyornis ?????00????????1220?????????????????????000111????0??
?????????000??1?????1000??0210??10???1?01111?2100?1???5?000?21??34???01?100?001
031110?011113003??0102?1??10212000????21023??3?12??10011??11?1213110???00?0??2
0?????00????0??00????21???????????0?????????????012300[12]0?11???????????10[01]1?1
11011101001020???101?1200111?101[12]?2110220101000010 10?11110112110111100101 10
111010000101[01]10112111123120110011?12001[01]0010?112110211110021111[12]2?1000
02?0??011?100?20???0110??1??00?00100?11??01??01???????????????????????????0?00?00
??1?0?????00???????????????????
???????0????0100?????0?00?0000?00?????????000?0???????00??000??0?01?0000??????0??
00111?00100?0?10201??000????01?0???????0????0000?0?0???0?0??1???00??0??1??????00
0??00100?0???001??01??2?0?00000?00000000????0000?????00?111?0?00001????10?1?0?0
?11?0??0????0??0????????????

Iaceornis_marshii ???
??1101?010311??
????113?03??????????????????????????????2??1??1???????21??????????????20???1??0???
??
???????????????21?02[23]11???1101010?11110112101???????????????????????????????1113
14011101110 20010??1?1?12110212110021??010
0?11???1???
??
??00111???????0?10????????????????0??0??0???
???0????????????????1???????????1????????????00????0???00???01???????????????0??????1??
?????????????1???0?00????????????????11?0??0????????????????????

Limenavis_patagonica ??
??
011?13???20????????????
??
??10000[01]010101011?11[12]3
13011?011??
??
??
???0?0???????????????????????????????????
??0000??????????
???

Lithornis
100????????????????01010100?00?111??2?02?00?0???????????????10000?00????02?101?1
?????????1????????2????????[67]?1????????????[12]??11000010311110011?13003??000210
101?20200102??2102?????12?01000???11?12130100?????0??200?01?2010???0?0?0???21??
?12????????????00???00000??300?11212100?00011111010110?11[01]1101?1111?01?101210
12?0111[12]1211122110100?0101101111011210111100110001[12]1110000101010101011111
13130110[01]11102[01]0120110?112110201100021111222100 10[01]?000011?1????????011
?111??00???????????01???1??
??0????????????????0???????????????????
??????????????????????????????0???????0?0?????????????????????????????????1??000????
?????????????????????????????0???0???
????????????0000???

Hongshanornis_longicrest
101????????????????11010?????0?11???0????00????????????????????000?001??00????1?1?1
0???0??0??01??1?0????????[234]??????????4???1??11?0111031110001111 30020?0112???01
?20????????2??23?23??2??100?1??????21[23]0100000000?000?01?1????????0?0????1?1?
0????????????0????0000?1?300???012??????????????????????????00??????1?????0?????1
10????????01?0??11???0?10???21000111???110???2????0????????????1??2000??000100?0
??0?0??000????????1???1100??[01]?00??2?0???1????????????????????????0100?11??001?1
1??????0???????????0??????????0??????0????????????????0???????????0??0??????????????????
????????????????0??0?0?0???0?????????0??????
??????0???????0??????????????????01?1?0?000?0?10???????????????????????0?????0?0???
00????0??100?00??0?????0??????????1???00?????????0?????????????????0??0?0?????????
0??????????00100?????????0????????????1???????????????

Liaoningornis_longidigitu ??
??3??1??0
???100?1????0?21211003000?????0???????????
??????????????????????????0??
?????????????011?01??????????0???0?????????????????????????????0?????01???2????????
?????????????????01?1?0??0011111110201000?00?????????????????????????????????????
??
??
???0????????????????????????????0?????0?0
???00????????????00??0?????????????????????????????????0??????????????????????????????
????????????0010??????????0??????????????1?????????????

Crypturellus_undulatus
10????012?020002?2001010100?00?111?120021000010111000 10???1?10000?000??102?00
1?1?????????1110111122100?01107?110??12?24??121?11010010311011011113003??00121
0001?20220112002102 3?23?12?010001001101213 01?0???01000200001?20000200?0?0010

21???120??01??????0????00000103002112121[01]01000111110101100110111111111100101
012101011211212111112?1010002101101011111210011111011000120110000111010101111
031401011111020?120110?11211020211002111122210010110??011?1010200000111111000
0??1100?10??01??11??
??????????????????????0???0??????
??2?????????????????
?????0??
?????????????0??????????????0??

Gallus_gallus
100???102?021002?2000010100?00?111?120021?0001?1000001????1?10010000000002110
1?1?????????101[01]1111221?0?01007?120??12?24??1[12]1?11012010311020011113003??
001211111?2?2001120021023?23?12?0100011011??213010010001000200100?20100200?0?
0???21???121??01???0??00???000001030011021212111112211011111111111101100011010
111210101120111211112110100021011111111121001111011000120110000101110101111
113140100121102 1?121111?112110202110021111322200111?00?0110001020?000111111?0
00??0010110??01??11???
???????????????????????0???0?????
??2????????????????
??????0???
??????????????0??????????????0???

Crax_pauxi
1001011?2?021000??001010100?00?111?120021000011100000100??1?10000000000002110
1?1?????????1000111122100?111?7?120??12?24??121?11002010311121011113003??00021
00?1?2?2001120021023?03?12?0100011011?12130100100010002001?1?20100200?0?0???2
1???121??01??????00???0000010300210212121111122110111111111110110001101011121
01011201112111121101000210111111111121001111101100012011000010111010111111314
01011211021?111111?1121102021100211113222001101000011000?020?0001111110000?11
100011??01??11??
???????????????????0???0??????????
???2????????????????????
?0??
?????????0??????????????0??

Anas_platyrhynchus
100???102?021002?2101001100?00?111?10002??00?1?100010?0???1?10200000100?02110
1?1?????????101?1111221?0?011?7?11???10?2410121?11011110311111011113003??001200
0?1?2?2001120021023?13?12?0100011011?121311?0???010002101?1?20100?00?0?0?1021
???121??????????00???00000113002102121111111221102111211111110110001110111210
101020111211113120100101010?1[01]11111210001110011000120110000101110111111123
140101011102 1?111110?111110102110021111[23]2210010210?0011?10?020?000111111?00

0??0100?11??01??11???
???????????????????????0???0??????
??2?????????????????
?????0??
?????????????0?????????????0???

Chauna_torquata
000??1012?0210002?101010100?00?111?100021?00010100010100??1?100000000??002110
1?1?????????1011101112100?111?7?11???1012412111?10000103111101111310 3??00121
01?1?2?200102?021023?23?12?0100011011??2130100100000000200101?20100000?0?0?102
1???121??01??????00???00000123001102121[12]1111022110111211111110110001111 0111
2101210201112111140101001010111110111211021110011000120110000101110101111123
140101111021?111111?1121102021200211112222001001000011000102000101111110000?
11101?11??01??11??
????????????????????0???0?????????
???2????????????????????
??0???
????????????????????????0???

Pedopenna ??
???
???0?0?0??0010??????????????????
???
???
??????????????????????????????00?000?????0??0????00????????0???11???????????????0??
???
???
??00????0???
00???????????00??0???????????????????????????????0??????????????????????????????????
?????????0?10???????????????????????????????????????

Epidendrosaurus ??0??1???????
?????????10??????????01?????0???????0??????1??????????????????????????????????001??
1??0??????001??????????????????????????????????????0????????0000030?0?0???0?????1?
??????????????1????????????????????????????3?0??????????????????????????????????????
????0???????0???????????????????0??0?0??????1???0?????????0???0???0????????????????
0??????000??????????????????????????100?00000??0?0??0?010????????00??????1????????
???0??00??
??????????????????????0????0??
??0011??????0????????????????????????????0??
???0?0???00?????????0?00??0???????????????????????????????0?????????????0????????0?
???????????????????0010??????????0????????????????????????????

Epidexipteryx ?01???????????????????1??????00?????0??2??0011??????
???????????100?0?0????00001020???10000???????????????0?[01]????1?1?123??10??0????
0101?010?0??????00?1???????????022?????011?03?0??????????0????011?0??0?10?000???0
0??1?1?0???00?0?00???0?0??????????1??2?????0???????2?0?????0??????????0????????????
???????????000?0????00??0?0???????????????0??0?0??????1???0?????????????00?[01]0????
??10?0?0?0????10?00???0?????????1??11
??1?????????????0?00?100?????0???????????0??????????0?????0???????????????????????????
??????????????????????????????????0????0??002
00??02??????????0???????0?1?????????????????????????????0011??1???0?????00??????????0
1?????????0?????0?0??????????0????0?0????0????????0???????????????????0???????00????
??0???0????0?????????0??????????001?00?????????????????????0???????????????

Incisivosaurus_gauthieri ?00?00?01?001??1??10001110100100 1?010?12000011000101
011010111121010000100 1?1?000210??00?0???
??00??00??01
?0??0?0?????????100000001000102?00010?100?????000000000010000 1??0000???000010
1?1100??0000???
???01020?00???0??000?0?
0?????????????11?1100000????1000000?0?0000?000?0?000???1000??1??00?0????000?110?
?010??01?0000?00000??00?00????0100??0111000????????????????001000000010000????
00200??02?1?000000000?????00110??
?????????????????????????????100??????????????0??0?001????????????????????????????0201100
???0?0?0??????0??????????000?0????????????

Citipati_osmolskae ?001001001001??2210001011111?01011?000102100011000
0100010001101211 20?0100010111?1?????????1011101100101211001??201??00220011 1?01
11200110100100?1100000100021 1001??02012020??10111111?2101000000000?000000000
00000000001102000000 0?0?001000?000002011???0???[01]00002000122000100000000110
2?1??0000??00000100 01010??00000200 11000[01]0?00?0010000101020??????0????111000
?0?0010000?000000000000?00100?0??0?0???0000000????00000?20000000000000000000
2?000?000000000020?000110??00[01]10?0110110000000 11?1100001????1000001?0?0001?
11000?001???1002??0???0?0????000?000??000??01?0000?0000000000100?????100??0111
000??????????????????10100000000 10000????10201???2?1?0000000???0???????????????????
??????????????????????10???00?00????????????????????????????0?0?0???00100????000?00?
?0???0?00?????????0?0?0?0010???0?00211??????????0??00?000?0???????0??????0?000010
0?0100????10?????????1?00?0?00011???100

Oviraptor_philoceratops ?00?0??????01?1????0??1??111?1011???0???1?0?11???01?0
???0?11?121120?01?00?01?1?1????????????????????????????????0????????????????12??1??
???00???10??00?00??1??0?????????????????????????????0????0????0??0?????00?00000???2
?00?0?????????????10000?0?0???????0??0???0????00???0?00????????10?0?00??0??00?00?1
?1????00???00?100????0????????01010?0?00?0?0????1?100??????1100???0????????00????

?????000?0??00000?????????????????????????????????????0??000?????20????1?0???02?0
??1101100???0011?1?????1????10????1???0?01????????01???????????????0?????0???00??0
?0??0??00?????00???????????????????0???1020??
??2?1?0000000???1???1???0?????????????????????
????????????????????100????000???????????0????????????????0??0???????211?????????0
0??????0??0?????????????????????00??0??????0???????????00??????????????

Microvenator_celer ???
????????21?20?0????????????????????011?0?11000121100???0?1?1002????????????00?00
0?11000?????0010002???0000?????????????1110??0101100?0000010???????0??0???00??11
?20?0??00???0?1??0?????????????????????0???0?2200000??????????????????????????????
??????????0?0001???????????????????20000?000?01?????00?0??010?00?00000000000001
0000?????00????????0???0??000002000000000000????????????10??0???????????????1?????
???0????????????00???
???
????????????????????????0???00??0001?01001?????????????010000?0?????100???????01?
01??0000????00??????????????0?????0?????????1????000?10?000???0010?0001????1??????0
0??00?10??00000000??000?0??00000??????????1?01??11100??001??????0001111????

Caudipteryx_zoui
00010???????????????0?111??10?0001?10???21000???0?0?????????????21120?0????????0?1?
?0?????00????0??00????1???0???01?????30?????0???0??0???01?0???100000?000?1??????0
201202???1????101?11??1??0????01000??1?00?0?000000?1102000??00?0?001000100000?
00????0???00?00000?1[01]2000000000????????????0??????0000????1000???0000?00?0000?
0?0??????????????20??0?0?000?11??00???????0?0???00?00?????????????1??0000???0000000
?000000000??0?0?00????0000000??0??2000000??0????????01101100??01?110???0??000?1
0???0000?????0????0?0?0001?00?????0?????????????????0????00???00??0?0??0??0????????
?000?0?????????1????0???00201???2?????????0???
??????0??0????????????????????????????1???????00??000???0000???????????????0?00??0?
0???001?0?0???00?0???0????10?????0???000??0?00?0???0?0???11??0????00????0?000?00
00???0?0?????0?0?00100???0?????????????????1?10??????1111???0

Ajancingenia
yanshini ?00?0?????????????????1?1?????1??????????????????????????????????
21120?01000?0111?1?????????????????????????????1???01????2?00???1011120011??00000??
100000100021??????0201202001101 1?11101101?0000?00011?000000000??0000??11?2000
?000?0?001000???????????????????0002?0??2200?1000000??1?????0?0??0??0000?00??1?1
0???000??0??1??0[01]0??02??????0101??0??0???0?0?0101000?????1100???00?0??????????
???????00????0?00?00?000?0?000??0?0?000?0?0?0??00?0????00?000000????????0110??00
?1?0?1101000??000?1??
??
??0011000?001?0?01??101000??0101?

?11??0??????00000???00?????1?000000010??1?1?0??????10???0???0?10?000????11???0??
?010000000100000000??000100?000000010??????????0??1?0??00?1???1??01011???000

Rinchenia_mongoliensis ?00?0????0??????????0111?1???1?11?00010??00???0000??
???00??1?12112??01000??111?1????????????????1??????????????0?????220?????????2??1?
??00?0???1??0001000?1?????1???????????????1?????????0????0??0?0???????0??00??001??
2?00???????????????0??????00????????00??210??????2??0120????????10?0?10??0???0?????
???????0??
??????????????00???0?00???002?0????
????????00?1?1?00001????1??0001?0?0001?11??0??01???1???????????0????000?000??000
??01?00???0?000000?0????????100??0????0????????????????????????????????0??????????
??1?1001??????????
????????????????????0???????????????????0?????????????????0????????????11?????????????
???????0???????????????????????0???????????0?????????????0?????????????00

Conchoraptor_gracilis ?0010??????????1???00111?1???1?11?000??21000110?00??
??1?0????121120?010?0?0111?1??????????01010110010??1?012?110??10????01?1?????200
11000010???????0010002100010102002020??101101[01]1?11010000??00010000010?000??
000?001102?0?0000?0?0010001000002001???????0?0?0200012??????000000000????????1
0???0100100????????000??0?010???
????????????????????????????00?00??00000200000000000?0000?002?01?????????0?020?
000??0??002?00???????????0011?1?00001????1??00?1?0?0?01?11??0??01????0?2????????
0????0?0??00??0?0??01?00???0?00?000?0?0??????100??0???00???????????????????10???0
???????0??0?0?
?????????????????????????????0?0????010?????010?????0?????0???????10???????00?0????
???21??????????????????0?000000???????????????0?1?00????????00????????????0?0??????
??1?00

Chirostenotes_pergracilis ?????1??01?01101??0???1?110?0??????????????????????0101
0??????21120?00000201???1????????????????1101?12????1?12???0????????????????101?1
??????????00?100021?001010221202011110?111[01]??01??100???00?100000200000??000?0
?1??20?0?000?0?00100????????????????????000?????[12]????10????????001????00???????
?????01?10???0????0011??00?????????????????20000?00000?????????????????????????????
????????????????????00000?00??00000?00?0????????000000?0???0000?????001020?00???
0?????????1?????????0?001????????????????????1?0?00???0????????????????????????????????
??1000001000??????
?00200???2?1?000??0000??????????0????????0?0??00???1000?0??????????????0??0??10100
0?????1?0100?0??0?00??00000?001?0??1?1?000?01?????1?0??????10?????000??0??????02
?1???0110?1???????000000000???00????00??00010?0??0?????1?0??0???????0?1??00???11
??00

Avimimus_portentosus ?00?0???10011?00??0??1?1???????1???1?????00?11??001

00110??????2?1???00?0??0111????????????01101011010 1?1?00??100???????????????????

????0100?????????00?211?01??0201202010?011?0?1000101?0???00011110020???00??0?0
?01?02?000000?0?001000????????0?????????00?01??0?02001????????????????????0??0???
00??0??????????02001000?????????????????????????????0?????0?0?0010000?000000000?
???????????2???????????[02]0?000?0000020?010000000000000??00?01??0??????0????????
????????????????????????1?0??
?????????????????????????????????????100??0?1100???????????????????0000001?0?00?
?????????????????????????????????0???????0001000001?????????????00001???????1010
01??0101?0100?0?000??001000??00???????0??0?1001??101????0000100000?????10011011
02?????101010000000????00?000???0010000000000010?0????1??01????0??00?1???1??0001
101??00

Falcarius ??01?1110000111101 0?????1????00??????????00??????00
00000??????100?000??0???????001010001?1??00011101012101000120001 00??0000??????
000001000100010?0000100?01100101022030101010010001000001000000011000000000
000??000?10?00?0?000?0?001000????????0?001???00?0?0?0??11?0??000???????000??00?
??0??00?00100?0?00??0000??0010000[01]0??????????0100020000?00000001110?1?0?0010
000?0010000000000010000???0100???0000000?000000000200000000000?00000000?0000
??00000?0?200000??0???00000?1100?00000000??????????????????000?00?00100????0??
??????????????????????????????0?????0??????????????01?00????00?00??????????????????
??????0000001010010????0??00000001???0??????????????0?0???????0001000001000??10
00??101000000010000000000?0101?010000000001?0000001000?0??00101000010?1?01?
11?10001001000000000011101002?0?101111000000101000100000??000001000?000000?0?
10?10000?011011002?00?10?00000010200

Beipiaosaurus
0??1???10?10??????????????10
01001?100????1??????????????000??0220?????????0000??0????0?1000000?0000??001???2
0220??0??0????0??00?0??00??????0000????0???0??00111???0?0??0????0010?0??????????0
??????????0???0??????0??????????????????????????????????????00????001000????????????
?00?00??????????????????????????????????10000???????????1??0100???000000???0??0??0??
???0???????0000000000???0?0?00000????????????10?????1?1100??0??000?1?0?????0?????
???????????????0???
???0??0?00?????????????????????0??????
?0???????????????????????1???0?00??00???00?1????????10?????0?0?????????00???????1?1
?0?????????1??1?????10???000?0?00???????????????????0??0?01000?01010???0?11??10??0
?1?0???1???????????????????1????00?????????

Segnosaurus_galbinensis ???
???????21??10?0000?000???0100?001????????1??????????1?0?0??????????????????0?10?1
?00000????0???2001110010202022 1??0110201101?11001?00?00?11000000021?0??00?01?1
??0?0???00?0?010?00??????????0?????????00???????0??????????????????????????????????0

?0????0??????????????????????????????0??????0????1????1???0010000?00100000????????
????????????????00?00000??10??????00?????000000000??21?0????00?????????????????
?2??????????0???
???
?????????????????????????????????????10????????????0?00???????21020010?0101??02
???????????0?00??????????????0??01??0??1??1????????2???????0??110????????1???????1
0100??1?1112221011111112?111201??????????0???1????????????????????????

Erlikosaurus_andrewsi ?0012???2?0?1?1??1010011100??0001?1000001000010000
000000??1111210010000020001?001001001??
???????0??0000?021?0??00??11
??00?0?00????????????0?00000010010??00000?000????0?000002000001???10?0100???000
0?0?10000??0000???1?0?0010000?0010000000
???00000000????????0000?0????000?????
000?0?????????0????01?0001100????1?10000?0?00?0?100000000???0??0?00??00?0????00
0001000000??00?0200?00000101000?00001?000??00?0?00???????????????00000001101?
???0???0100100020000000000000??????0????????????????????????????????0?00???????
??????????????????????????????????0000???????00??0???0?011????????2??????????????
11210?11???????1?0?1????????????????????????1?01001000????01??????????010?0?01??0
??????

Alxasaurus_elesitaiensis ??
?????210?100???????????1001001??????????0?01010000?1?0101002?0?1???????????0?00
0??0?10?0000120?11??0??1?20?21???11?????????1????0?????????00000?10000??001?1??0
0?0??00?0?0???00??????????0??????????0?????0????0???????????????????????????????????
0??00????0??00??[01]0????????????????0????????0??11??01??????0?0???010000?????????
??10?0100???000000???0??0?????????????????????????????1000000?0????????0??????????
???????????000???
???0??002
0?????????????????????0????????????11101?????1??0?11??0?00??010?0?????01?????????2
????????????????200??0????1?1??0??0????1??1?100???????0000000??1??0???0?00???11?0
????01100??11?1?????1111?????1?00???10?1???0????????????1???????????????

Neimongosaurus ???
??????????21????0?????????????0??001?1??000?110101?1?0?10?2?1?1?02200????????000
1010000??????????????111001?2?????????????????????0??00????0??000000021?00????0??
1??00?0??00?0?????0???????????0???????????????1[12]200?0???????????????????????????
??????????0?????00?0000?0??????????00000200?0?00000?0?11001?0?0?10?0???010000?0?
00?????????????????????0????????????20?00???????0?00000000?0210??????0????????0????
??????2?1100?000??
???
0????2???????????????????????0???????0??1??11???????0??01?10?0?00???????21?2????????

?????2?1???1?00??00000?1???0??00????00????00??????100010?2???????00?????1???0?011
1?111001011????1?1121?????111111????10????1??11??0?0??0?100???1?1??00?????0200

Erliansaurus ??
??10??1??????????1???????????????0?0???10
?0?0???00000????1?0?1??????????????2??1????10??0?0?0001?0??00??????????00????????
?????????????0??????????????????????0??????200??
??????0????0????????????????????????????????????01?0?0?10?0???010000???00010???????
100???000000????????????20?00???????0????????????????0??????????????????????????????
????000??
???
??????????????0????????????????????????????????0?0?1?0?01????????????????02?0???1?00
0??????????????00100???????0??????????00???100?????00?11????????????????011?111111?
?11?1???0?1?11111011?0?????????1???????????0?1??????01??0??????

Suzhousaurus ???
??0101??01000??0??1????0??????????00101
0000????????????200111001?2020?2?1001002011010110 0?00??????????????????0????????1?
???????00???010?????????????????????????0?????0?0??????????????????????????????????????
?????????20010000[01]????????????????20000?00000?01010?1?0?0010?0???0100000??00?
??????????????????00?000?00?1020000????????????????????0????????????????????????
??2??
???
????????????????????????????????????1011110010000 0?11010?00??????021020010?0101?
00201010???????????????????0???0??01??00?1????1000?0?2???????00?????????????????1
1?100011????1112212111110???????????????????1?0??01102?0?2??????????????????

Nothronychus ?????1112??11?1?0?0?????????????????????????????????0
0000??????????????????????????1????????????10101?10?01?1?00?100220?001?????0000
0?100?00????00000200??10?1?20202211001002011 01?1100100?1000110000000 21?00???0
0??????0?0?000?0?010?00????????????????????????0????1[12]200???????????1??????????????
???????????????????0010000[01]0??????????00000200?0?00000?0211001?0?0?10?0???010
000???00010???????000???00???00?000000?1020??000000000?00000000?0210??0?000?0?
???000????????0???1100?000?000??
???000?01100
??????????????????????????????????????0???????0?0??1010110???0000?110?00001?0?0?0
21?20010?1101??02???1?10000?0?000?10?????0?1?0000010?0??1?11?1?0010020000?0000
1110?1?2??????1?1110?0001???1111222101 11111?12101120??0????1?1?0???10?????1???10
?01??????200

Enigmosaurus ??
???

???????????????111?01?2020221100??0201101??????????????????????????????????1??????
??00???010??
???
???????00?000000?10???2??????????
???
???
???????????????????????????1??1?????????????????????2?020?10?1101??????????????????
???????????????0??01?????1??1?12212
1100??

Nanshiungosaurus_brevispinus ??
?????????????????????????????????1???????101?121???0????????????????????????????????
???
?????????????????????????????0?????1???2
00??0???
???0??2???????????????
???
???
????0??????????1???1??0????????????????????????21?2??10???01???????????????????????
?????????????01??0??1????????????????????????????????????1??????????????11222??11??
???

Therizinosaurus ??
??0010?000
0????0?00200?????????????????????????????????????0??0?1100000021?0???00???????????
??00??
??????????0??????????????????2000??00000?01?1001?0?0?10?0???010000?????010???????
100???00000??????????????????????????????????????0??0100???????????????????????????
?????00???
???
??00?1010?00100101???????????????????????????
???????????????001?0????????0??????????10??0000????????????????????????1110111111?
???????????????2?111?0?????????1??01????0?????????????????????

Haplocheirus ?0010???00010?0?12011011101112001?000020100?0?101
0??0001?0010000010110110?1000001101010100??10??0?0111201??0?00001?0???0?00????
???100210001210?0?00010???001?00?101002010010?011000?00000000?0000?0000000??0
0?1110001100010001?0???0010?01000000000??0000001?00000?102000000000???00???0
0???0???000000??0??0??0000?000?000????0?????????????20000?000000010101??????100
???000?00???????????0???00000??0000000?00?000100200000???????000?0??0???0100?00
0???0?0??????????00?0??????????0?0000001?00001??0?0000000?0?01000000?0??11???0??
0?01??01?0????000001000000??00??100?00000000000?000????00??0??0?0??????001?????

???0?0??0?0000????0???00000??00?00000??00?????????000???????0??1???????????00?10
10100??0100?00?0????????0?01??0???00?0?????0?????00000?00?101000??00?0??010?110
01001000100000110??00??0?101???00000???0000????????000????????00101???0?1?1?01
0?????0??0000??1???????????

Alvarezsaurus_calvoi ???
???????????????????????????????????????01000???0???0?20?0?2012?????????????000?00?
???????0??1?0000101?00?2??????????????????01?000??0??0??11000000??00????????1???0
?0??00?0?????0??????????????????????0????10???
???????00100???0????????????????2?000?????0?0?0?????????????????????????????????????
???????????0??000?00???2????00000000?000???00?02???0?????????????0????????0??????
????00???
???
?????????????????0????????0001?00????????00?10?0?0????????????000?000????????????????
?0??00?????00?????????????0??0??0????????1000???????10?????101???????1????00???????
??00000???????1???0?000?10???????1???0?0??0??0?????????????????????

Patagonykus_puertai ??
??1?01?112????012010?2?2???????????????00210
???210?????1?0???1????1010?????20????21202??11000000000001210??0????001??0??????
?0??????????0????0??????????????????????0?????01??????????????????????????????????????
???????0?00100??????????????????????20000?000?00??????0?0?0000??0?0010000???0???????
???1????????????0???0????10020000000000??00?0???????10??0??????????????????????????
??????????0???
???
?????????????????????????????????00001?01100?0?????000?1100??????11???0?0?01?01?1?
000?10??1????0?00?????0????00?000?0??1???0??1001?101??1?0?1111111????????????0?0
0000??0?????00??0000?00?0?00??1?????????10??1???0???0????1??????1???00?

Achillesaurus ???
??020???2??????????????????????
???????????????10??01?2????????????????????1??????0??1?01000??0???00????????1??????
?????0?????0????????????????????????0??
???
???????0?????????????2????00000000?0??0??0??
???
???
???00?0??????????0????????00?0????
0???????????????????????????????11?????????????????11??????????????0?????????????000????
???1???0?00??????????????0?????0?????????????????????????

Mononykus_olecranus ??????00???????112??????????????????????????????????

?100??????????????????????????2????00???????1?1?1110200l???2?10?2??????1???1000?000
200030210?02201100??1????1?201000?2?????3??0??02101001110112120000300000001??00
???1??0?0??00?0?0???00??????????????????????????00011?????????????????????????????
?????????????0?0010?0?????210???00?????2?010?0?0?0?010?011?0?0001000?0010000000
00??0000???30000???0???0??00???????2000000000000??0000??0?00?0??0??000???????01
?0?????0?0????????11102??
???
?????????????????????????????0???????1001001000????????1010100000100?????011???0
???01?010000010101?100000100????0??????00??1??0???021?111001011111111101011???
????0100000000000?000?0000??000010000?000?10????????100?10100?0?01???1??00011?
????0

Shuvuuia_deserti ?00101002?010111201100010ll?01111?10020100001010
010010???1110?0010100100210 0??0211??000??011111011102100?0?201012012110100?1
000?00020003021??0220110000101001?201000?2002203??03?021010011101121200003 00
00001100000110000001000 0?00??00100000000l?010??000000000?000100000000?101000
?10000110?0111000??0[12]0????0100?0010000[01]0?0210?0?00?????20010?000?01010101
1?0?0001000?00100000?000?????????30000??000??00?000?00?10200000000000000?00000
00?00100000?0000020?0001?01?1010001???????1110200?000?000??0?1??0000?0?00000?0
0000100???0??0?00??01?0????0?????0??000??00?0200?000000??0?0?0??00?000?001?0?0?
??????????????????000000000????????00000?000?0??000000??00??????0?0???????000100
10001011?0001010100000100??0?01110000???01??1??????????0???0????00001????1??00?
?1??00110210?110010?1111111??0?01002?0??00??0?0000000?000000000??000?????00000
010101?0??0?00110????00?1101?0????????????

Parvicursor ??
??10??????????1??2????????????????????????
??????????????????1????2?1000???02203??03??2??100011?11211000030??00?????????1????
??????0?00??0??????????????????????0?????0???
????0?00?00???
??????????0????????1?2000000000000??000?000?00?0???????????????0??????????????????
??11???
??
?????????????????????????????1000?????00??????????????????????????0???01??10??00?0?01
??000??100????????????00??1?????????1?111????????????11100010?????????????0???????????
?0?????000??00000000010??????????0???1?0????1???1?????????????

Albertonykus ???
???
?211?????1?0???030?????????0???????????
???
???

???0??02?
??
??
??1????????????????????????????????0???00????0?
???????????????????????????????????11???11??1??
??0????0????????????1???????????????????????????????0

Albinykus ??
??
?????????????????0??????????????????????????????????1101??121100300000?????????????????
?0000?????0??
??
???????0??????0????????000000000?0000000?00??0????00????????0???????????????????1
1??
??
???0?0100
00??00?????????????00??1??
?????????00000?10???????????0???????????1????????????????00

Bonapartenykus ??
??112??0?????????????????????????????000210
???????????????????????????01?????????????212?2??1?????????????????????????????0?????????????0?
0???????0?????????????????????????????????????0???
??????00100???????????????????????20000?000?000??10???
?????????????????????????100???????????????????????????????0???
??
??
???????????????????????????0?1000??1?????0?010?010???????????????????????0?0?01?????????????????
???????00?????00?????????????0??????1???011??????????????????????????????????0??????????????
??????0?????0??????????????????????????10????0???????????????1??????

Ceratonykus ?0010???2??10???????????????????11???10?201?0?0?0100
??0???????????????01?0??????????????????????????????1?1?????????????????????????????????10????0?
2???0??1????????212?0003???00????????????
?????????0?????0??????????????????????0???????????????????????????00???????1????111????????
????????????????????????21?????0??????0000?0???01????????????????????????0????????????????????
?????????????????????????????????????00000000??000?000?00?0??????????????????1????10?0?????
??????????0??0???0??????????????1?0????0?????0??000??0
??02???0?0000??0?0?0??00?000??0????0??????????????????????0??0?00??????????0?0???????
??????????????????????????0???????0001??
??0?0??1000???????0??????????????????????1?????1?00?0???????????????1?????????????????????
?????????????????????????00?00001?????????????01??????????????????????????????????0

Linhenykus ??
???111?101?1??1?0???2010020?2???????10?0???1??
????????0?21110??????????2??????????????????0?1010001????2?2?0?030?0000???0????1?
?????????0?????0??????????????????????????01??????????????????????????????????????
???????0?0012?0?????210????0?????20??0???0?0?0???????????????????10000???0????????
??31??????00??0?????????1?2000000000000??000?000?001?????0?0?????????1???????0???
??????????02??
??
???????????????????0???????0001?01000?????0??????1????????????????????????0?0??00
?0?0???000???00?0??????1??0????????????1011????????1111?1???0??????????????0??????
??00???????????01??00?00001???????????0?1?110?0???????????????????00

Xixianykus ??
???102?00?21201??0?????????????????????
??????????????0?01?1??1?200000?20022?3??03?02?0110111011121211??3???000??????11?
??????00?0?00??0??????????????????????0???
???????0?0010000???
????????????00?000000?1?2000000000000??000?000?0?????????????????????????????0???
??
??
??????????????????????????????1000?0?????????????????????000110000???01?010??00?0
?00010?0???????????????????????0??1???1011????????????111??11?????????????0?????????
?0?0000??000010000000001??????????0????100????1???1??00011001???

Nqwebasaurus ?0????????????????02????2010????????0100?00??1????
?0?00??????????????????????????02????1??????00?11001???1???????????????????????????01
02?000??0?00?10110???????????????????????????00?????0?00001000100000000?000?1??00?
?????0?0?0?????0????01?00???00?????????????????10200??????????????????????????????0
0????????????????????????0??????????????20????0?0???0101?0??????10?0????????????????
???????00000??00000??????????00????????????00?0????00??0?0??00000????????0??????0
?0??????????0?001??????????????0?0??????0???0??????????0???0??????0?????????????????
?????????1??????????????????????000??0??0?0??????????????????????00????????????????
???????????????????????????0??????????????????????????000??01?0?1?0?0???????????????
?????????0????00?0???00?????0?111?0???1??0??012?????100??00010000100001???????0?
????????????0?????????????????0?000010??????????0???????????1?1???0?????????0?

Shenzhousaurus_orientalis ???0???????????????21000??10?0??1???000??00?000?0????
???????0?00000000??0??0?1?12?0??1????????????000??1???0???00000???0??????????????
????????????010000000??11000100101?000001100??00001?0??????????????????0??1??1?0
011?????00???0010??10000010????????00??0?00???0???00?000????????????????????00???
??0??????0?00?0?100?0?0?0???
???????0?????0?00000?00000?0002?000??????????????????????????00?????????????????000

00????????????01?00??00000??0?0000?00?0?0000000000??00???0??0??1???1?????????????
??10???????0?????????????????00?0
0??00?00?0????0?????????????????????????000???????00????????????????010100000?000
1?00??0??????????????????01?????0?10??00??1??00?2?0000????????100?0????????00?10??
??00????????00000000???00??????????????0???0??0???1???????????1???0????00?????

Ornithomimus_edmonticus ?00010?1101101?101021000?010101011000000000000000
01000000??????00000000010?1001?1???????0?001?1011000011100010100000000100100?
?????0112001200000?2020010000001100011010110000011001000001100010001000002 0
??00?011111110121110000?0?0010001000000001?????0001?000000002100000000????????
?0?0?10???000010110??????000000010000[01]0?0???????????????0??0?0?0?0?01?1001?0?0
0?000???0000000??00??????1??00000??0000020?000?00000200000000000?0??0?????0??
0??000?1???????0???0??0000000????????00010000?00000?00?0000?01?0?0100000??0??00
???0??0001??01?0????000001000000??01?00????0000101?0000??000000??0????0????????
1??????????1?????????????????00210???0?00000?010?????????????010?00?00001000011????
????????????????10?00???1?????????????????????????????1???????011110011111111?11000???
????100??0???00?01????101?0???1??0?0000?10000000000????000000000000000100?0??????
??1?????????????10?01????????000

Archaeornithomimus_asiati ???
????????????????????????????????????00?1011000011100001?100000000?0??????????010200
?20000?0110[12]0???000?0110001101011000001020100000 1100??000?0000020??0000?10?
?10??1?1??00??0?001000???
??????????????0?001000?[01]0????????????????20?00?00000?0?0??01?0?0010000?00000000
0000010000???00000??000??00???0000000200000000000?0000??00?0?10?000?????????
???0???????0?0????????00001???
???
????????????????????????????????????0???????0??1?00001?00??00000?00020010?010?010100
000?01011?00000000000?000001000?????001?101?001?11?0????0000100000001100001??0
11???????110?0000011000000000000??000000000?000?10??????101?0?1?000?000????1??0
000001?000

Anserimimus_planinychus ???
??1??????????????????0???????????1?200?2
????00?2020010000001100011010 1????001?001????????????????????00002???00????11??10??
1?1??00?0?001000???
?????????????????????????????????????0?????????0??1??
01000??00000???????????????????????????10000??00??0??0??00?1??????????????????????????
??????001???
???
??10?0??1?1??????????????????????
???1?0????????????11011011001??1?0?????????100???01?00??????????????????????000?10?00

0??????????????????????010???

Struthiomimus_altus ?00010?110??0??1010210002011101?11000000000000000
10??0001?01??0001000001020001?1?????????001?1011000011100010100000000100100??
????0112001200000012010010000001100011010110000001?001000000110001000100000020?
?00001111110121110000?0?001000100000000?1??????00?1?0000??2210????0???????????0
?0?1????000?????0?00???00?0000?0000[01]0?0?????????????200?0?0?000?0101001?0?001
0000??00000000000?100000??00000??0000000?000000000200?00000000000000??00?021
000000?10022000000?0??0?00?00?????????0010000000000?00?0000?01?0?010000000000
00???0??0?01??01?0????0000010?0000??01?0000??0000101?00?0??000000??00?0?0??????
?????????????1000011010?00????00210???0?000000000????????????00??00?00001000011
?????000000000200?110101?10100100?01011?000000000000010001000001110011111111?1
?110000??0000100000011000010001101 00???1??0?00001100000000000???000?000000000
100?0?0?101?1110000?0000?1010????????????

Gallimimus_bullatus ?00010?1101101010102100020 11?01011000000000000000
10000001?0100000000000001020001?1?????????0011101100001110001010000000010 0100??
????0112001200000??2010010000001100011010110000001100100000011000100010000020?
?00000111111012111 0000?0?00100010000000 01?????0001100000000221000?000000010000
00?0000??00000?0?10200??1000000010000[01]0?0??????????????20010?000000010 1001?0
?0010000?000000000000000100001??00000??0000020?000000000200000000000000000?000
?021000000?1002??0000?00??0000000???????0000100000000000??0?0000?01?0?01000000
000000???0??0?01??01?0????000001000000??01?0000?00000101000?000000000?000?0?0
00????????????????0010000111100000???00210?0?0000000000000000????????000000?0000
1000011[12]0000000000000020011?0101010100100?0101100000000000000000100?100011101
11111011001?110001??000010000000110000100011010 0???11000000011000000000000??000
0000000000010000?001010111000010000?1010?000??11?0??

Garudimimus_brevipes ?000?????01101????02?00020101000??000000000000000
00?00001?01000000000000002?001?1???????0??0111????0001?10001?100??00?????10?????
???????????????????????0000001100?????????????0??001000001?000?000?000001000000?0
1??1110?2?1?0?00?0?0??0001000000000??????00001000000010???00?0000?001000000?0000
??000001011 0000??000000000 10000???0???
??????????????????????????????00?000000000200000000000000000000000?021?0????0000??
??00???0??001000????????00???0000000000002?0000?00?0?0100000000000???0??0?01?
?01?0????00000100 0000??01?0000??000000000000000000000??00?0?000??00?010???????0
010000000010???0???00210???000000000000000?????????0?0?00?0000??00011100000000???
??????????????010100100?0101?0000000?000000100?10001111??????001000?1?0001??000
0???????????00001011 0100??????000??????????000000??00000000000000010000?0010??01
??0011??00?111?00000001?100

Pelecanimimus_polydon ?00????????1????????2100?2?1??0000??000000?0000????

??????????0?000?0000???????000211??0001??000???0?0?111?00???????????????0??0?00??
?120????00??0?2010??1001010?
???0?????0?????0????10000??0?1?0????00????0000?0?10???0?0??????????0?0????????????
?????????00???0?10??????0???????????????0??0?0?0???0?????????????????????????????????
???1??00000??00000???????????????????????????????????????0??000???????????1?????0?0
0???????????001?00??00010??0?0000?00?000000000000??00???0??0?01??01?00?0?000001
1??000???????????????????????????00??0?000??????????0010000????0??????????????????0
??00??00??????????????????00000000?10???????0?0???????????????????????0?01??????????
???????????????????????????010?1??111??????0????0?2?0?????000001100??????????0?110
1??????????0?000??????????????????????????1???0??????1??????????111??1???????????

Harpymimus_okladnikovi ?0??????????????????2100??????0??????000000000000???
???????????000000?0000????1?1200??1???0???1???0?00??10001?100000?00?001????????0
?1?0012000000000010?10??00????????1????????????00?00??????0??0?010000010??0000010
11????1?0???00?0?0???001??0000??1?0????00??0000??11?10000000????????????0???????
???????0000??00?00000?0000[01]0?????????????????????????????10??01?0?0010000?00000
0000000???0?010?00000??0000000?0?0000?00?????00000000?00000?00??11??0000?0????
????0???????000?????????0?001?000000000????0????0??0?0?????0000?000???0??0?01??0
?????????????????????0???1?00??????0?????????????00000??00?0???????????????????????????
????????????00210??000????????0??????????????0???????000??0000?1??0????0000??2001?00
?01?1??0010?????????0??????????00?0??0001100100011??0??0?11?00????000???00000100
0????011???0??01???00000110?000000000???0?00???0?000?100?01??1?1?01??00??00???1
???????????????

Beishanlong ??
??0102001
20000????0100??????????????10???1??00??????000001100?10001000?01??000?0???1???0??
1?1????????01000??????????????????????????????210??
???????????????0??0????????????????20010?0000000101001?0?0010?00?000000000000010?
?????????????????????0???????????200000000000?00000?00????0??0?0?0?????????0?0???????
?????????00?01???
???
???0000020011?????????????????????00?
????0?00000?0???00?????00??1?0???0??1???????????1000000?1??00?0?011??????????????00
0100??00??????????????0000000001??????????1???10?0??0??0???1??00?1111??00

Sinornithomimus ?0001?????1?0????1?21000??1010001?000000000000000
000?000??0???00000000?00????1?1?????????0001?0?10?0???100?1?1??0?00?0?0?100?????
?010200120000?011010010000001100011010110000001?00?00000110001000?000?010??00
0001011100121?1??00?0?0010?01000000001?????00001000000?0210000000???????????
?00??000001?1?0??????00000001?000?0?0??????????????20010?00?00?0101001?0?0010000
?0000000000??01000010?00000??0000000?000000000200000000000000000??00??1100?0

000?????????000???000000????????0?0010000000100?00?0000?01?000100000??0?000???
0??0?01??01?0????000??10??000??01?0000??000??????00000000000??00?0?0?0??????????
???????10000????????????00210???0?000000000????????????0???00?00001?000?????0?0?
?000000200?100?01010100100?0?010000?000?00?0??1?0?1?00111?1001110?1001?11?00??
?00001000000110000100?11??00???11000000110?000000000???00000000000001000??0?
101?011?000?0?00?101?????????????

Qiupalong ???
??
???????????????00??1100????????????01100?????????0??0001000002???00????????1??????
??00?0?0???0??
????????0??
????????20?0?0?00000?????00000000?0000?000?02????????1????????0??????????0???????
?00???
??
??10100?00?0001??????????000?
010001??0??????????1??110????0?????????????????????1??01??????????????????????????0
000??000??0000?00001??????????01??????1???????1?????????00?0

Kinnareemimus ???
??
??0???00001???00????????????????
????0?????0??
????????????0??
???????0?????????0??????0000000000000?000?02??0
0??
??
??0???????????????000??00
000?00???00???1??????????????????????????????
??0???0000???001????????????0??????????????1?????????????

Huaxiagnathus_orientalis ?00?0??????????????0001???1???0?????00??0?????????????
????????00?0010????????00010?1010?00??0???1????????0??????00???0?002010????00000
0000?00??010000000020?0??000100201?000012?0???0??????0??0?010000000?0?011??000
????00?0??00?0?0010?0100000?0?1?00????0????0000??200???0000????????????????????
??????????????00?00?1??0?[01]0?0????????0000020?00?0?0?0?010100??????10?0???0?????
??????????????010????000??00?00??00000??????000?0??00??0??0????100000000????????
000????00000?0?00??000000?01??00000??0?00?0?00?10000000000???1?????????????????
?????????00?00????????????????????????????????????0?????????????????????????????????
???????02?00???0??????????????????1?0?00???????0?0??0000??????0??0001100???1?0?000
??00000??0?????????????0?????0?0???0?0?0?00?101000??01?0?00?0?00001000?000??0????
???0?????10?????00????0?0?0?0000????0???0????0000100????????????????????000??????

????????

Sinosauropteryx_prima
000?0??????????????0001???1???0?????0000??????00??0???????????00??01???????1?0001
001010?000?01??100????1?0?0????00?110000201??????00000000?10??010000?00020????
??0100201?000?11?0?1?00?0??000?0?0100000000?011??0000??000?0??00?0?000??01000
0000?1?00?00?????000?0??20000?000??????????????0???????0101????????0000?0??0?00[
01]0?0??????????????0?00?0?00??010?000?????10?0???00?00????????????1??0100???0000
000?00000??0?2?????00??0??00??00?0???0100000000???????000?10???000?????????0000
?01???0000??1??1????0?1?0?00??0??????0?????????????????????????00??0??????????????
????????????????????0???02?00???0????????????
?????000100???????0?0??0010??????0??00111?0???110?000??00000??0?0???0??0???0??0?
00?0???0?000?00?11100???0000?00?0?0?001000?0?0?0??????010????1100???0000???0?00
0?00000??000???????0000100?????100?????????????000??????????????

Compsognathus_longipes ?00?0???????????????00101?1??00???00000????000????0
00???????0?00000100?1?0010000100101010 00?01??1010???1?0?0???000?0120002010????
?0?000000?10????0?000000??????0?01002010000012?0??????????00?000100000000000011
00?00????0?0???00?0?0010001100000001?00000000?0?000??020000?0000??0?0??0??????
0???0000????01?0??0000000010000[01]0?0????????00?0?20000?0?0?0?010100????0?1000
???0???00??????????????010????0?0??00??0?000?00??????00??0?0000?00000?0010000000
0????????00????000000????????00000?001?001000?1?0100?000100000000000??0???0??0
?000?00?0????0000000?00?0??01?12????????000???????11?000?????????????????????????
0??????????????????02000?00000020000?0???????1?0000???????000??0000?0????0???????
01???1????00????0??0?0?0???0??????0??0000?0???0?000000?101000??00?0??0?000?0010?
??0?0????0????10????1100??0000????0???0?00??????00???????0000100?0010100??1?0???
?????001??????????????

Juravenator_starki
0000???????????????00010??11000?1???00000?0000?0?????00?????0?0000010???0????000
100101??00??0???1???????0???????00??100002010????000000000000????0000000000?011
00????????????????????0?????00??????0?0??000000?10?000?0??0??0??00?0??????01000000
0?1?00?0?000??0000???20000?000??????????????0????????????0??????000?00???00??0?0?
???????[01]00002???0???????0101000?0?0010000??000?000?000???0?0????100???0000?0?
??00?000??2??00??????000??0?0000?00100000000????????00???00?0?00???00??0?0000?0
11000?00?01?0100?00?1?00000000000?00???0??0?00??00?0????0000000??000??01?10???
?000??00???????11?000??00?0????????00?????????0??????????????????02000???0?????0??
?0???????0?010???????????????????????????0000??00??1?0?000??????0????????0??????0??
???0?0???00000?0??101?00??00?0??0?0???0????0?0?0??0?0?????0????0?0?????000???0?0
00?0???????0??????????00100?0?????00?1??????0???00???????????????

Sinocalliopteryx

0000???????????????0001??0???000???00?000?000??0??????????0?0?000001???????1?000
10010101000?01??10?????1?0??????00??1000020?0?????0000000??00??0100000003?0?0?
?000110201?000012?0?????????00???0??000??0000?011??000?1??0000??00????010?01?00
0000???00?0000???00000??20000?0000?00?????0????00?????0????????????00000001000?[
01]0?0????????00?0?20000?0?000?010100??????10?0????0??00???????????1??010????000
0000?0000000002??00?0????00?0??00?00??0100000000????????00??10?00000???00??00?
000?01???0?010?0?0000?000??0000000000???0???0??0?00?????0????0????00?00?0??0???
2?????00????????????11?0????0?????????????0?????????0???????????????????2?00??00?00??
??????0?????1???0000??0??000?00010??????0??000010????1?0?000??00000??0?0???0????
??0??0??0?0???0?000?00?100000??01?0?00?0?0?0010?0?0?0????0?0???0??0?0?00??0??0??
?00?000?0000????00????????000101?????10???1??????????000??????????????

Mirischia ???
???0??0?0??????????????????????????
?????????????0????????0001?02000???012100100010110????0?????????????1????????????
?????????0010??
???????0?1?0??
??????????0???00?0?000??
??
??
??????????????????????????0???000??????????????????????1??????0?0?00?000?1??0?0?????
?????????????????0???????0??0????0????0????????????1????????????????????????????000?0
????00??00???????????????????0???????????0????1??00??001????

Ornitholestes_hermanni ?0002???0?0?00?1???0?010?01110001?100101?0??1000001
?01011????0000001000?0?000010?00101001?????011?0101210000??000?0010??1????????
???????01?00?????????00001??1?0?0100101000001??0?1???????0????????00?00????00000
?0010?00?0?0000?0?0000?01000000001?01?00001000002?112000000010?0??000000?0?00
???0000?0?0000???0000000010000[01]0??????????????????????????????????0?0?0010000?0
00000000000??0??0????1000??0000000?000?00000?0000?????????0000?000?001??00?0??
??????00??0???0001?0?????????00??00?1?0000000000000000?000000000??00??0???0000?
000?00?0????0000000?0000??01?0000?00?0000100??00001?000?0001100?00000?01000??
???000000000000???0???0200000000000020000?00000?00000000???????0001000002?10?00
?0??????10010?01??000000000???0100????000????0?00?0000?0000???????????001??1010
?0000???0???0????0?00??001000000?00???000000???000000??000??????????0?1000?1001?
0001????0??0?1010?01???????????

Coelurus_fragilis ??
??????????????????????????????????0??01001000012100??????0?0002?????????????0?0??0?
10001????0???????????????????????????01100?000001?0?0000110?0?0????0?00??00???0???
???????0?0????0???????????????????????????11200??
???????001000???????????????????2??????????????01000?0?0010000?000000000000010000?

??0????????0000?????????0020000000000000??000???????????0000???????????0??????0???
????????0???
??
??????????????????0???????000010000 2?????0?000?0??100100???????????0?0001?00000
0000?0000?????0?????????????1??00??????????0000??000???0??00?0100????????11000000
0000?00?????????00000??00000001?????????0?1?0?11221111?????????????????

Tanycolagreus ?000????????????????00?0??????0???00110????????0?00?
?????????????????????10?00??????1?????????????0011?000?????0????0?0??0???????0000
00000011?1000000???????????????????????110010000011000000010000000000000??00?
??0??000?????0?0???00?????????????????1???1????[01]2000???0??????????????????00??
0?0??????????0?00001000??0?0?????????????20000?0000000101000?0?0010000?00000000
000001000000??01000??00000?????????0020000000000000000000?00100?00000???????
?0?0?????????????????00000?????001000??????????????????0??????021000000?000?0???
??????????????0??0??00???????0?0?000??00???
???????????????????????????0??????????????????????00001????????0011001001000000??
????????00???000000000000000000?000?????00100000000??0??0?0?100?1000000000000 1
01000??????????????0000000000?????????0?000000000000010?????0??0?100101221011???1?
????????????

Tugulusaurus ???
??0????????????????????????
??????????0?0??????????????????????????????000?0?100??00110?0?1????0?????0?????????
??
??
???????????????????2000000000000??
?01???
??
??00????????????????000000000000?0??
?????0?????????0?????????????????????????????000001???1??????????????????????????????
??????0?????00??????????????????0001??1????1??00?00??0???

Zuolong ?0000??????????????0000010111?0???000?00000011000
00?????1000??????????????????00010?101??1?010001000????1???000000?0000??????????
???????000?0??????0?????00?0100??????????????0110011000011 0?00?0???00010?0?00????
?0?0100??????00???0???0011000000??0???00??1???0??0002000??0000??????????????00??0
000000?????????010?0?10??0?0????????????????????????????????0??????10000?0000000
00000010000???????????????00?0?0?00?0020000?????????0000?000?0?1???0????????????
??0???000100????????0??00?????0000000000000?00000001000???????1???0??00?0000???
???????????????0?????0000?00000?0?000??10??0010?0001101?00000???????????????????
????????????????????????????0???????????100???????00000??0???000???0??????00000????
?0?00000?0?0?0??0000000000???000?000?00??0????0?????000??00?0???0???????????000

011?????0?0??0000?0?00000??00???0?0???0000000????00??0?????1?0?10??000??01?0?01
??0000001?000

Bicentenaria ???????????00?1??????0?0??????00??00?????????????00??
??????????????????????0?000000001????????????100?????????0????????????????????0?0
0???????????0?1????0??10????????????????0???00000?10???0?110???????0??????????1???
?0?????????????????00??0???0???????????????1???0??????????00????????0??????00??????
?????0???0010????0???????????????2???0???0???0?????0?0?001??????0000000?0????????
??????????????0??0???0????2000?00000000?????????????0?????????????????????0???????
?????????00????????0????????????0???00??0???????????????????????????????0?0000000???
????????????00000000?1??
1?2?00??000??0??????0???????????????????????1?00??0????00????????0??0?????????0000
?02???00?0???????00????????0?????00??????0???00???0???????00???00?????0??????00??0
0?0??????0??0?????00?????00????????????0?????0?0??0?1?0?????00?????????

Kileskus ???????????????????0?0112011l???????????????????????
?????????????????????????00000?101???
???00?00??0?????????????????
?????????????1?0000?0???0??00??????1????????0??001?????????????????????????????????
???
???
???????1011201???000?0000?0000?0??
???100010???
??????????00???0
0?????00???????????????1??????0?????????????????????????????0????????????????????????
??????????????????1?????????????????????0??????????????000

Guanlong ?00020?000000?010[01]10001120111 2000?00110000001
0000000000?10????00000010?00??2000000 1010?100?000000000111000000000000?0???0
0???????000000100000?01000000030000100001002000000001110010000011000?00110000
1000000000000000000000000?0?001000110000000 1100?00?11101102?1120000?0010????
0000?000?00??0000000??0?0????000000010000?0???????????????20000?0?????0101?00?0
?0010000?00000000000000010000???01000??0000000?0000000002000000000000000000000
?00100?0000020?20?000?00??0001000???????0000000000000112010?1000?00?1000001010
000?003??0000??0??00?100010000001100010??01?1000?00000000000000?1000011?000000
?00000?????????????020?01000100000000000????0?000000010???????010100???????0000
10000210000?0?000001000100000000000000001000000?0110000000100000?0000000?00011
00000100?010010?1000100000000000001000000000?00100100000000000000000??000000
000000010100???1?0011?1001??1000001??0??????????

Sinotyrannus ???????????????????0?01???101????????????????????????
?????????00??0?1????????0?0000101??????????????0???????????????????????????????????

???????????0??00??0?0?000??0??0??
???00?????????1?0000?0??100??0?????11????????00?001????????????????????????????0?
??
??????????00?000?0?0???0????????
???00??????01?2?1??10???0??1?00????1??
??0?????????????
?????????????0???0001???????????????????
?????????0?0??????0?????????????0?1???????????????????????????0???????????????????
??00????????????????

Proceratosaurus_bradleyi ?00?2???????????????00011?01112000?00??????????00?00??
?????000?00000010?00?12000000101011???
???00??0????0?
0??0???????????110000?0?1100?00?11?0?1?2??????00000100000?????????00???0??00??10
?0????00??
???0???????0?????????
?????????00?0010112?10?1000?00?1?0000101???0????????????????0?1000100000110001?
??????0???0???00000000??0?0????????????0000??000?????0??0?0??????00100?????0001??
?10?00000001000?????11000??
??????????????????????0?00????????????1????0?0???????????????????????????0??0010??????
???????????????????????????????????1?0?1??????????????????000???????????????

Dilong_paradoxus ?0002??000000???0?100011??1112000?00110?000010000
000100???????00000000?00??200000001010?1?0000?0001?0??1000?????0??0?0?0??0?????
??0000001100?????0000000?00??1??00110201?0?0001?00?000?0??00??00010000100?0000
?00000?0?00000?000?0?0010001000000001100?001111?110201?20?000000????00000???0
?0???0000010110000???000??00??00????0??????????????20000?0?0?0?0101000?0?0010000
?0000000000000?0??0????1000??0000000???0?000002000000000000000000??00?0?1000?0
000?0?20?00???010000100????????000000000010002?11?1000100?0?000000000000103??
0100?00??000101111000011000 10??00?1100??00000000010????00000?000100??????????
?????????020?010?010?0????01001??10?10002?00?11?????01110000000100000100001????
???00000110001????10000?0001??000???01100000000000??010000000??11000????1?01?
01001?????00??00???00???00000?00100?00?0000010?0?000000????00??0?000?0001?100?
??1??10112????0??0000???0??????????

Eotyrannus_lengi ????????????????????000120??12??0???110????????????0?
????????0?000?0?0??1???2000000101012?00?????01?0??1???????????????????????????000
000010????????0?0??0???00000?0?00???00?0???
?00?0?????0?????0?1?000?00??10??0121???111?0?211?0000?0???????????????0??00?0010
0???00??0???0?0010?0??0???????????????200?0?0?000?0?0?0?0?0?0010000?0000000?000
0????????????00???????????????0????????????????????????????????0???????????????????????
?1????????????0?00?????1?00101???????00?0?01????01000?1010??1??0?00?00????????????

????0?????????????????????01?1??0??????????????????001??????0?00??????????????????
?000?1002110100??????????2??00???00???00????0??????????00??011011?000???????????
???????????????01??00?00100?000?0???0??????1?0??0?00????1000??????0?00????????000
0?00???0000100?00??0??????????0000???0?1????0??????011??????1???0?1?1???????????

Juratyrant ???
???????????????????????????????????0?00?0000001?10??00000??0????????????????????
??????????????00?00?0100001101011000011?01???0??10????0????????????????????1???
?????00???0010???????????????????????2????11??
??????0?00100?0?0??
????????????00?000000000?0?00?000??00?????????????1?????????????????????????1?0?????
??
??
???????????????0????????00?100011?00100?0????????????????10000001100100O?????1100
001?????????????????????0??01??1??1?????000????????????0?????????????0000?0????????
??000000??000??0000??????????????1??1??????11??0????1?????????????

Xiongguanlong ?0002???00000?????1?000220???0000?001101000?2100
0000?0001??000???????????????00000?101??200000000000111000?????????????????????
?????????????????????????0?12?01000????????????????????0000110??????????????????0?????
?0?00?????000?????????1?000000???00?0?1121?2102011???0??0000?00?000??????00???0?
?000?????????????00?00100??
????????????????????????00?0000000??20000???????0??????????????????????????????0???????
000100??????????????0010?10001????1?00?01?0?01?0000100??0010?0100??0??0??0?????0
???01000?0???0?0100???001?111110010????100?1?11??00?100????????????001001000010
?1???????????????????????????????????2?000??0000000011000011????????????????????????111
??001???????0111012?????????????????00??0????????????1??1?010?10??????????????00??????
000?0??00001????????????000000?????000000???????10????1??????????1???0?0?01??01?00
01????

Dryptosaurus ?????????????????????????20????????????????????????????
???????????0?0?0??2???????0000101?1?????????????????????????0???00??????????????????
?10???????10?0?????????????[01]?0?0?2???????101?0100110001000100??2???0?????????
0?0?????????0?0???????????????00??????????????????11????0??????????????????????????????
?????0??????????????0??????????????????????????????????0?0?0010000?00????????????????
???????????????????0????????00200000000000000??000??0?????????????????????????????????
???????00?01?????????????????????????0???
???2
12?1?1??10??????????????????????1?0???01
2?1?0111??????1????0??????0??????????????0?????????????????000000????????????????00?1
???00??????????00??0000?00001????01???????????0?1??00?0?0???100001O???

Appalachiosaurus ????????????????????0?0?201110??????120??????????????
???????00000?000?0????????00001010?2????????????????????????0?0?00?????????????????
??????????????????????????000010120?10???10??0100110001000100000 2?0?00??????0???
?0????????0?00100?11000000?1???1?021??????????????000??0??????000?????0??????????
?0000???0?????????????0???
????????????????00????????00200000000000?0000?000?00????????????????????????????
?????????00?????1????????000?000?01?010111?101??1?0020?01?10??0?0????????????????
???000000010100010?00?????????????????11?1
10??1?????????0??1?????2?0??????????????????????????0??????????????????????????????1
?01110???111111111021?0000?????????????0?????0?0???00???????????0000000???00??0???
??0???????????????????00?0??00000001???0???????01?????1??00000??100???010200

Bistahieversor ?1102?????????10?2200002?01110000?001201000?211000
00??????????00000000?20??2?000000101012??0?????????????????????????????????????
???0???????????????2????????10??00??
00?0??0????????????120000011?00?00213?0?112??????0000000?????????10??0????00001?
??0?0????00???????????????????????????????????????00????????????????????????????????
??11??????????0??????
?????????????0?1011001000?1010101?010111010101010020100010 01???10000??00000020
00021100000 10000??1111111?1?1001011001211111?01000?010011111??????11?111101?101
11111100?11212101100112?10??2?00????111??????????????????0111?1???????????????????
????????011?????01101111?120010000?????????????1??1?0?0???????????????????????????0
??0???11001??????1??????????000?11???????????

Albertosaurus_sarcophagus ?1102??000000?10022000022011100000000120100002110000
00?00010000000000000000 20??200000001010120????0?10?001?100?0????000??0????0?????
??000000010000???011001031200110000000101200100 0110??0?00?1000??0010000020000
0001000000000000?000?0?0010?0120000001100 0?002131011121020110000000 0???00000
100?00??00000100100 00??000000000100 00?0?0????????0000020000?00000?0001001?0?00
10000?000000000000 0010??0?????000???00??00?00000000020000000000000000000?00??01
00000??020100?000??0??001100?0110?0000001011101100 10000[01]1001[01]10010111010
2011100211[01]1[01]1101?001000000100010201002001100100011111011111111101011001
111111000[01]000111[01][01]111100001011011111111011111110011121211 1100?12?10?121
011???????1????????????????1111102011111?102111111021 1101??1??1???111111111 1220
10000000??00000101?011010?100010?000??00?000000100?000000??0?00??110??0000000
0??0000?00000000010110011110101 11????010000001 1???????????

Gorgosaurus_libratus ?11020?00000011002200000220111000000001201000021100
0001000100000000000000002001200000001010120?000001000010100 0000?000000000000?
00001000000000100000001011001031200110000000101200100011010010001000?000100000
2000000010000000000000000000?0?00100 0120000001100010 0213101112102011000000000 00
00000100000??0000010?10000??0000000010000[01]0?0????????0000020000?00000?0001

001?0?0010000?000000000000001000010??1000??0000000?000000000200000000000000
00000?0010000000020100?000100??00110000110?000000101110110010000[01]100101001
01110102011200211 01[02]1?0100010000001000102010020011001000111110111111111010
1100111111100010001111111??0011011011111110111111100111212111100112?10?12101
1???????1????1101????1?0?0111110201011111021111010211101?011?1?2?111111111?2201
000000010000001011011010?100010?0000100000000010000000000000?0000110?00000000
0??0000?0000000001011001111010111 10??0?0000001100?00????00

Alioramus ?01020?000000110022 00?0?201110000000120100002 11
00000100010000000000000200?20??000010101?00000001000011100000 0?00?00?0??000
????????????????????????????120011000[01]001012?010??????001001100??0?01000?02?
?000??10??000000?0?0000????010??1100000011000 1?0213101?12102????000?000000000
00100000??00000100?0000??00000?00100?0?0???
????????????????????????????????????00?0???000??2000000000000000000?0??0?1?0????
0020110?00???0??0011000???????0????00111?2????001110111110?011101010012012 1001
021110101110001000010200001000000101101112011111111001011001111111?1011100110
1110100011011011110110111011110011121211 1110??10000???0000010000 11110?1012210
11111?????????????????1111010??????1?1111121??00?111?12?0100000??????0???0?1?1?01
0??000????????????0????01?0000?000000?0????????????0000??0??00???0?000?1??1101111
?1?1??1011??0?000?1101000000000

Teratophoneus ?1?0???0???????0??2??????0111?00????1201???????0000
???????????00??000??????????000010101??1???
???????????????????0???????????????????????????????????0????????????????????????????0????0?
???????????????1?0000?01?????????3?????????0???000????????????10??0???00?001???0??
???0???1????????????????????????????????????
??100?????????????????????
?????????????????????0????0?01???????????????2?021?0001??10??????????0???01?1??????0?
??01??1?1??21?111?100111101211?????????????????????????011?1?120?1????1??1100?11?
????????11????????10????????????1??????????????????1?01???1?????????????????????011?1?
1????????????????000???????????????????0?0??????????????????????????????0??0??????????????
????????????????????????????????1??????1??????????00??1????????????

Daspletosaurus ?11020?000000?10022000022010100000000120100002110
00001000100000000000000200120000000101012010000010000 1?100?0????000??0????0??
?????000000010000???111001031200110000001012001000110??0100?1000??00100000200
000001000000000000000?0?0010?0020000001100010121310111210201100000000???000
00100?00???000010010000??000000001000 0?0?0????????0000020000?00000?0001001?0?
0010000?000000000000010??0????1000??000??00?00000000020000000000000000?00??
01000000?020110?000??0??00110000110?000000101110120011020110021110211110102 10
1201210101[12]111111111111101101020000211001011[01]1121121211111100?111012211
1111101110011011111[01]100111110111101111111111100111222111101112?1001210110001

1101111021111???????111111?211?111?112111??1??111?1??1????2?11????11?12???00000?
?11000????1??1?010?1000???000?1000?0??????0?000000000???0011000?0???????????????
?????????111011110??1??????????000?11???????????

Tyrannosaurus_rex ?12020?01100011002200002201010000000120100002110
0000100010000000000000002001200000001010120100000100001010000000100000000000
?0????000000010000???1110010312001100000010120010001101001001100010001000000
20000000100000000000000000?0?0010000200000021000101213101212102011000000000
000000100000??000001001000??0000000010000[01]0?0????????000020000?000000000
1001?0?0010000?000000000000010000????1000??000??00?000000000200000000000000
000000?00100000000020100?000?00??00110000110?0000001111111200110210 1[01]0[13]01
102111101021112010??1012101111011[01]1101111021010211001011111211[12]12111111
1001111212301111111111101101111111111110110201111111111121122212110111121111
1211211111101111021111211111111111021101111121111102111111112211211111111111
1221100000001000000101101101 0?1000100000??0000000001100000000000100001100?10
000000??000000000000001011101111010111101101000000110100001 0?00

Tarbosaurus_baatar ??2020?011000?10022000022010100000001201000021100
0001000100000000000000020012000000010101201000001000010100000?1000000000000?
??????000000010000?011110010312001100000010120010001101001000100010001000002
0000000100000000000000000?0?0010000200000021000101213101212102011000000000 0
00000100000??000001001000 0??000000001000 0?0?0?????????????20000?00000?0001001
?0?0010000?000000000000010000????1000??0000000?000000000200000 0?000000000000
00?00100000000020100?000?00??00110000??????00001111111200110210111301102111101
021012010??10121111111011111011110210102110010111112112121111111000111212301 1
1111111110110111111111111101102011111111111121122212?1011121111121121111 11?111
?1021112??1??1?11111102110111?11211111102111111111221121111111111112211000000010
000001011011010?1000?000000100000000011000000000000100001 10??10000000??000000
00000000101110111101011110 11??00000011???????????

Anomalipes
zhaoi ??
??
??????????????????????????????????????121101???????????0?01?????????????????????????
??
??
?????????????????????????0??????????0??????????????????????????????????????0????????
??
??
??1000???00100?00?????0??
??????????????????????????????????????1???11??
0?00??0????????????????10?0??0???????11011001111

New characters added to Lamanna et al. Matrix

231. Femur, greater trochanter, anteroposterior width: less than or sub-equal to anteroposterior width of femoral head (0); greater than anteroposterior width of femoral head (1)

232. Accessory trochanter: present (0), absent (1)

233. Accessory trochanter: minor (0), significant (1)

234. Notch between lesser trochanter and greater trochanter: present (0), absent (1)

235. MT III proximal end: wide (0), mediolaterally narrow (1)

236. MT III, distal articulation, medial hemicondyle, transverse width, sub-equal to transverse width of lateral hemicondyle (0); much less than transverse width of lateral hemicondyle (1)

237. MT III, anterior flange near proximal end: absent (0), present (1)

238. MT III, longitudinal groove on distal articular surface: absent (0), present (1)

239. Femur, posterior trochanter: present, developed as a slight tubercle or flange (0), present, hypertrophied into a ‘shelf-like’ structure (in combination with development of the trochanteric shelf) (1), absent (2)

240. Metatarsus, proximodistal length: greater than 44% of length of tibia (0), between 44-38% of length of tibia (1), less than 38% of length of tibia (2)

241. Femur head, shape: subspherical (0), anteroposteriorly compressed (1)

242. Femur, femoral head: orientation: medial (0); dorsomedial (1)

243. Femur, accessory trochanter: confluent with lesser trochanter (0); separate from lesser trochanter (1)

244. Femur, femoral head, posterior displacement: absent (0); present, femoral head more posteriorly positioned than greater trochanter (1)

245. Femur, greater trochanter, anteroposterior width: less than or approximately equal to anteroposterior diameter of proximal portion of femoral shaft (0); greater than anteroposterior diameter of proximal portion of femoral shaft (1)

246. Metatarsal III, proximal articular surface: rectangular (0); triangular (1)

Lamanna et al. Matrix

Herrerasaurus_ischigualastensis

0000000000000000000000000000000000000000000000000000000000000000000000
00000000000000000000000000010?0000000000100?0000101??0000000000000000100000
000000000000000?000?0000110010100??000??0000101??00??0000???00?1000?000000?00
000?1??00112000?001

Velociraptor_mongoliensis
0001000000001010010100101000100000000010000000010000100000000000000000000
0000100001100000000000100101101111011010111101-
1101011110111000011111120100111000100110000000000?0000000011000?0000??00100001
000000000110011?000?000000000000?1?000010100?011

Archaeopteryx_lithographica
000?10000000101101001100010001?10002110?0?1??0020????00????0000001?1???100001
021000011-
0100000020100??0101?00?110110012111001111121011011010111020101?0100?1101200?0
000?0?0?00000001000?0000??001?000000?010010?0?111??00?00000?0000?0?1?0???0000?
????

Incisivosaurus_gauthieri
100110101000011?0111210011?01?00?0000?10010?0??200111?1?10?01??111?0101?210111
020??00011??11?111011???0
0000000100?????????00100000000000000???????1???????00???01000?????????????????
?????

Caudipteryx_zoui
10?110?01??0?1110?0?21?01?10???0110001110100??????????????????0????????0??1111?2
???0001??????11?0220?????010????0?1??10???1011?1020100?001220110?021100???10001
21??0???0??0?00?001000?000?00000000?01000?0010???0101????0?00000?000000?0001??
??0??0?00

Avimimus_portentosus ??????????0??????????????10110??1?2121201??00?
101111?1???????????????0?1??111??01?????11000???21?21000110?110??0???2?02100111?
1?????00000?210?10020101001111222100010000?0???????00??00?00????0??0000??0??10
110??11110????????0?0?0001000100020000010

Microvenator_celer ???
?????????????????????1111?2????11001???????????2???0??1?????????0????1?1011??1???
?0???112?11?112???11101????????0101???001[01]?????00??0000000?0??????0?1??1???0??
1?0???1?????0??00???0000????2?01100?

Oviraptor_philoceratops
1??????????1?11?????21????????1??1000???0????????????????????????1?????????1?12122?
?10011???1??1??22???0??1????????????1???101101?211????????????????????????????0??

?001001000000??0??1????????????????0??????????0??11?0?0???0??00???????0??????????

Rinchenia_mongoliensis
11122?0111110111???1211111111?11110101110011111?1111121?11111112111111111121
2?01100111111112122111001 1?1010010102?12101000??????01101121101???111111????
??1101?001011???????00111??????00000001????????1?10???1110?0?0?0???????????????
??????0

Citipati_osmolskae
11?2221?1111011?11112111111?111?1111011111??????111????11?1?11111????1?11?111212
2011001111111111122???????1????????????????0?101?2??????????????????????????????11
011001011?01010?0??1100000000000000100????010100?01110?001000100?0??1?10??02
000?0?0

Zamyn_Khondt_oviraptorid
1122221111110111111211111111111111101111111111111111211111111112111111111111212
2011001111111121221110111?101001010210210101101121110100121100111211111111100
0101101?001011?0000010??110?0000000000?0100???00?1?100??11???001?0??00??0????0
???????????

Khaan_mckennai
1022111?1111011?11212111111111?1100011100?111?111111??0??1?????1??????111111212
10110011111111??1221110??1????00?0102??210??1101021110?000110001?1211111?11000
1010011001001001010011111 0?000?00100000100000??01?1100?1?10101000?0000[01]011
?100001100?010

Conchoraptor_gracilis
10121111111101111121211111111111110001110?1111111?11112111111111121111111111?1
2?01100111111112122111011 1?2010010101112101111001211101000210001112111111110
0010101?????????110???1??11????????????????0??????11?00?011?0?0000011?0??0?1?00??
021?????0

Machairasaurus_leptonychus ??
??2??????????????????????????????0012????
????????????????????????????????????111110??????????????????????00??????????????????
????????1???????????????????

Nemegtomaia_barsboldi
11120111111101111111210111111111?110001011011?111111?1??11?11111112111111111121
2?111001111111?1121221110[02]11?201????????????????????????10002??00?1????11??????
???10111101011?12?11??111100000000100000001000??0??1???1??11?1?1?0001?000????????
??????????

Heyuannia_huangi ??????????????????????????????????1??????????????
???????????????????????????1??2121??1001????1??????21???21-
12????1?????121?11000002?20???01?1100111?1???????????????1?1?1011112?2101?1?????
????0?00000100?0???0101?00?1?11?0???0?00001??1?00????101??1?

Ajancingenia_yanshini ???????1???????????????11?????1?11000111?01
11111??111??11?1??111?1???1111111212?0110011111111112122?110011?201001010101210
110000120200100121000111211111111100010?011?101011112121011111000000000000000010
0?00?0?1?100001?1??0???0?00001011?000001001?010

Gigantoraptor_erlianensis ??
???????????????????1121111202011100111112????1?2????????????0010?0??00?00000?11?1??
1???????????1??1111111000?0???0001010000??????????0???0001001110100???0??????1??
???110??112?10???10?11?2110000111

Caenagnathasia_martinsoni ??
?????????????????????21?1?1????12???????????????2???????????????????????????????????
???????????????????????????0001???0????????????0111111?1???????????????????????????
????0???????1??0???02?00?01?

Elmisaurus_elegans ???
???
????????????????????1[01]1????????????????????0?????????????????????????1???????????
?????????????1????10?0???????0

Leptorhynchos_gaddisi ???
??????????????????????21?1?[12]????1[12]???????????????2??????????????????????????????
???????????????????????????101??????0??????????????????0111121?1???????????????????
??????????????????1????10?0???????0

Chirostenotes_pergracilis ??
??111??????????10?????0????1??11
??[12]1101???11??1?1100110????????????000101000????????????????12?1111???????????
??????????1000?01010?020001??0

Caenagnathus_collinsi ??
???????????????????11210111020112001111111112??2??????????????????????????????????
??????????????????????????????00010100????????????110112101111111???????????????1?
?010??201??????0000????2?01100?

Anzu_wyliei ?1??101100?1100??????????????????0?000??????10
11??100?1??1??0?011?????1121011?0201120011111111?121221110211?1?10010?02?021000
10????1110???1?2?1?1102111111111?????1?000010100000??01?0??0?10112111011111??111

1?110101101?00101021201110??010????1?001?0?

Hagryphus_giganteus ???
??0010???
?????????????????????????????????????000010??????????????????????2?????????????????
?????????1??????????????????

Elmisaurus_rarus ???
???001?????
?????????????????????101?????????????000010?0??????????????????11???10????????????
?????????1??1????1000???????0

Nomingia_gobiensis ??
??00?1?01?00101020??????????????
??11101101111101111111??????????????????????1?00???????????????????11????0???1??
?1????????1??10???????????00????

Epichirostenotes_curriei ?????????01?010??????1????????????????????????????1
10?1?1??0??0?1?111??1???2?????????????????
???????????021?????????????????????????????1?????????????????????111??0?0???1?????
??????????10?????????????????

‘Caenagnathus’ ??
???1121111102011200111112?112??2???
??????????????00010100????????????11112111111111???????????????1??010???0????????
???????????????

Macrophalangia_canadensis ???
??
????????????????????0011???????????????????0???????????????????????????????????????
??????????0????????????????

Banjilong
11?210111???0111102121111111011??11101????0??11?1111????????1111121?0111??11?212
1??000110??1?11?122?1??111
11001011??????????110??????0?00000????????????????00?0?000?0??????????????????????
?

Caudipteryx_dongi ??
???01?0?????????????0010?1?201
00?011?2011000211?????100012????????????00?001000?????????????????01?10?????0??1
?????????????00000?0?01???????????

Ganzhousaurus_nankangensis ???

?????????????????????1[01]111?21?[12]011001?????111???2?????????????0???0???????????
???????????????????????????0001????1?001?11???????1????????0?001?10?0???????0????
???????111???0???????????0000???????0

Jiangxisaurus_ganzhouensis
1???????????????????????11???????1?001????????11?????????????1??1??????111111??101?
1?0011?111????122??10?????????1???????1?1100?0?10????0???????????????????????????1
?001?10??2?????????????????0?00000?0?????????1?0??????????00??00??????????????????

Nankangia_jiangxiensis ???
???????????????????11101?2?????100????????????2??????1???1??1??0?0011001?????????
01110111111121100111 1????????1101??0???????1?00??11?????0?1???1????11???????01??
?11?0???0??0?00??1?1????1?00??0?

Ojoraptorsaurus_boerei ???
???
?????????02???1??????????????????
?????????????????????????

Shixinggia_oblita ???
???1?2?1??1?????????????????????1
10000000?????1????1?????????????????????????01?????????????????????????????????????
?????????????????????00?0??

Similicaudipteryx_yixianensis ???
??0?11?0?1?0??1?2???????1????????0?0
01?21110?????????0??0?12???????????????????00??????????????????????0??????????????
?????10????????????2000???0

Wulatelong_gobiensis
11?2211?11?1?1???02121?111?1??1?111001??00??11???????????????1111????????????????1
01????1??11????12???????1???100????2??????????1??????1100?211111121???????000?01?
??????????0??????011????????????000????1????1?10?101????????????0??0????1????1????
10

Yulong_mini
1020101110110110111121010111010?01000111010?111110110??0????????0?????111111121
220110011[01]1111?12122?100??1????001?10??[01]??0000101?21010?110?10??0??0?1?0?
?11?001010?11001011?11000?100110??????0?0000010???????1??????1110?01000??00????
????????0??????

Anomalipes_zhaoi ???
???

????????????????????1110???000?0???00??????
?1?????????????????100?112110110000

New characters added to Funston and Currie Matrix

251. Accessory trochanter: present (0), absent (1)

252. Notch between lesser trochanter and greater trochanter: present (0), absent (1)

253. MT III proximal end: wide (0), mediolaterally narrow (1)

254. MT III, distal articulation, medial hemicondyle, transverse width: sub-equal to transverse width of lateral hemicondyle (0); much less than transverse width of lateral hemicondyle (1)

255. Mt III, anterior flange near proximal end: absent (0), present (1)

256. Metatarsal III, longitudinal groove on distal articular surface: absent (0), present (1)

257. Femur head, shape: subspherical (0), anteroposteriorly compressed (1)

258. Femur, femoral head, posterior displacement: absent (0); present, femoral head more posteriorly positioned than greater trochanter (1)

259. Femur, greater trochanter, anteroposterior width: less than or approximately equal to anteroposterior diameter of proximal portion of femoral shaft (0); greater than anteroposterior diameter of proximal portion of femoral shaft (1)

260. Metatarsal III, proximal articular surface: rectangular (0); triangular (1)

261. Femur, greater trochanter, anteroposterior width: less than or sub-equal to anteroposterior width of femoral head (0); greater than anteroposterior width of femoral head (1)

262. Accessory trochanter: minor (0), significant (1)

263. Femur, posterior trochanter: present, developed as a slight tubercle or flange (0), present, hypertrophied into a ‘shelf-like’ structure (in combination with development of the trochanteric shelf) (1), absent (2)

264. Femur, femoral head: orientation: medial (0); dorsomedial (1)

Funston and Currie Matrix

Herrerasaurus_ischigualastensis

00000000000000000000000000000000000000000000000000000000000000000000000
0000000000000000000000000010?0000000000100?0000101??000000000000000000100000
000000000000000?000?0000110010100??000??0000101??00??0000???00?1000?000000?00
000???????000?0000?000?1?00110001??20

Velociraptor_mongoliensis
00010000000010100101001010001000000000100000000100001000000000000000000000
00001000011000000000000100101101111011010111101-
110101111011100001111112010011100010011000000000?000000000011000?0000??00100001
000000000110011?000?0000000000000?????00000000?000?

1000010011??00

Archaeopteryx_lithographica
000?10000000101101001100010001?10002110?0?1??0020????00????0000001?1???100001
021000011-
0100000020100??0101?00?1101100121110011111210110110101110201010?0100?1101200?0
000?0?0?00000001000?0000??001?000000?010010?0?111??00?00000?0000?00??????00000
000?000?

10?0?00?????0?

Incisivosaurus_gauthieri
100110101000011?0111210011?01?00?0000?10010?0??200111?1?10?01??111?0101?210111
020??00011??11?111011???0
0000000100?????????0010000000000000???????1???????00???01000???????0??????????
?????000?

??????????????

Caudipteryx_zoui
10?110?01??0?1110?0?21?01?10???0110001110100??????????????????0????????0??1111?2
???0001??????11?0220?????010????0?1??10???1011?1020100?001220110?021100???10001
21??0???0??0?00?001000?000?00000000?01000?0010???0101????0?00000?0000000?0????
??000000?000?

001?????00?0??

Avimimus_portentosus ??????????0??????????????10110??1?2121201??00?
101111?1???????????????0?1??111??01?????11000???21?21000110?110??0???2?02100111?
1?????00000?210?100201010011112221000100000?0???????00??00?00????0??0000??0??10
110??11110????????0?0?000010?????1000000?00??

00100000101020

Microvenator_celer

???1111?2????11
001????????????2???0??1?????????0????1?1011??1????0???112?11?112??011101????????01
01???0011?????00??0000000?0??????0?1??1???0??1?0???1?????0??00??????????????????
????00????000?0021

Oviraptor_philoceratops
1??????????1?11?????21????????1??1000???0??????????????????????1?????????1?12122?
?10011???1??1??22???0??1????????????1???101101?211?????????????????????????????0??
?001001000000??0??1????????????????0??????????0??11?0?0???0??00???0???????????????
000???0???????????

Rinchenia_mongoliensis
11122?0111110111???121111111?1111010111001111111?1111121?111111121111111111121
2?0110011111111121221110011?1010010102?12101000???????01101121101???111111????
??1101?001011????????00111??????00000001????????1?10???1110?0?0?0????????????????
???????000?

?????0???0????

Citipati_osmolskae
11?2221?1111011?11112111111?111?1111011111??????111????11?1?11111????1?11?111212
20110011111111111122???????1????????????????0?101?2???????????????????????????????11
011001011?01010?0??1100000000000000100?????010100?01110?001000100?0?0??????00?
0??00?000?

110??000?0??20

Zamyn_Khondt_oviraptorid
1122221111110111111211111111111111011111111111111112111111111121111111111212
201100111111111212211101111?101001010210210101101121110100121100111211111111100
0101101?001011?0000010??110?0000000000?0100???00?1?100??11???001?0??00??00????
???????????000?

??000?????????

Khaan_mckennai
1022111?1111011?1121211111111111?1100011100?111?111111??0??1?????1??????111111212
10110011111111??1221110??1????00?0102??210??1101021110?000110001?1211111?11000
101001100100100101001111110?000?001000001000000?01?1100?1?10101000?0000100????
??00000000?000?

11000000101?10

Conchoraptor_gracilis
10121111111011111212111111111111110001110?1111111?11112111111111112111111111111?1
2?0110011111111121221110111?2010010101112101111001211101000210001112111111110
0010101?????????110???1??11????????????????0??????11?00?011?0?0000011?0??000?????
00000000?000?

100??0???0??2?

Machairasaurus_leptonychus ??
??2???????????????????????????0012????
???????????????????????????????????111110?????????????????????00??????????????????
????????1???????????????????????

??????????????

Nemegtomaia_barsboldi
1112011111101111112101111111?11000101101 1?111111?1??11?111111121111111111121
2?11100111111?1121221110211?201????????????????????????10002??00?1????11?????????
10111101011?12?11??11110000000010000001000??0??1???1??11?1?1?0001?000??00???????
???????000?

??????????????

Heyuannia_huangi ??????????????????????????????????1??????????????
???????????????????????????1??2121??1001????1??????21???21-
12????1?????121?11000002?20???01?1100111?1????????????????1?1?1011112?2101?1?????
????0?00000100?0?0?0101?00?1?11?0???0?00001????????000000000000?

100???0?1????1

Ingenia_yanshini ???????1????????????????11?????1?11000111?0111111
???111??11?1??111?1???1111111212?0110011111111112122?110011?201001010101210110 0
001202001001210001112111111100010?011?101011112121011111000000000000000100?00
00?1?100001?1??0???0?000010?00000000000000?000?

10000000101?11

Gigantoraptor_erlianensis

??1121111202011
100111112????1?2????????????0010?0??00?00000?11?1??1????????????1??1111111000?0???
0001010000??????????0???0001001110100???0??????1?????110??112?10??????????010?00
0??011?01111101111?10

Caenagnathasia_martinsoni

???2101?1????12
0??????????????2?1002?1?0?1????????????????????????????????????10?1???????????0001
???0????????????011?111?1??????????????????????????????0????????0?????????????????

?0???0001?1?20

Caenagnathus_collinsi

???1021011102011
200111111112??2??????????????1???????????????????????????????????111101?001?????00
010101?1??????????110112101111111?????1????????01?0010??201??1????????????0???0??
000?00????000?0021

Leptorhynchos_elegans

??12111?1????12
00???????1????2???1111????0001
???0???????0????0111121?10???????????1???????????0?????0??????1?????1111001111111??
1??10?0???0????

Elmisaurus_rarus

??????????????????????????????0???
??????????????????0??10?????????2????1?????011??????????????????11?1???1111??0?????
????000?10?0???????????????????11????1?????????????????????1??1111111111011120??1
1??1000???0????

Apatoraptor_pennatus ???

???????????????0?1???12111110201110011111111112??211002111?????????2?011100100111
0111???101???????????101?????????00010100010?10??????01?11?11111111210??????1?10
1?11??011??101?01????111????????????1001??????????????

Chirostenotes_pergracilis

???1121111102011
2001111121112??2????????111??????????10?????0????1??11??21101???111?1?1100110???0
0010100?100101000??0111121111111112?1111????????1??010???01??1000??11110000110
01001100010?00??0?120

Hagryphus_giganteus ???
???0010???
??????????????????????????????????????000010??????????????????????2?????????????????
?????????1?????00??????????????1???????????????

Anzu_wyliei ?1??101100?1100??????????????????0?000??????10
11??100?1??1??0?011?????1121011?02011200111111?121221110211?1?10010?02?021000
10????1110???1?2?1?1102111111111?????1?000010100000??01?0??0110112111011111??111
1?110101101?0010102120 1110???1??1????0?????000000??????0??110

Nomingia_gobiensis ???
???00?1?01?00101020??????????????
??1110110111110111111111??????????????????????1?00????????????????????111???0???1??
?1?????????1??10?????????????????????

0?????0???01?0

Epichirostenotes_curriei ?????????01?010??????1????????????????????????????1
10?1?1??0??2?1?111??1???2?????????????????
???????????021?????????????????????????????1?????????????????????111??0?0???1?????
??????????10??0?????????????????????????????????

Banji_long
11?210111???0111102121111111011??11101????0??11?1111????????1111121?0111??11?212
1??000110??1?11?122?1??111
11001011???????????110??????0?00000????????????????00?0?000?0???????0??????????????
???????????????????

Caudipteryx_dongi ??
???01?0?????????????0010?1?201
00?011?2011000211?????100012????????????00?001000?????????????????01?10?????0??1
?????????????00000???????000000000????001???????????

Ganzhousaurus_nankangensis ???
?????????????????????11111?21?2011001?????111???2????????????0???0?????????????????
??????????????????????0001????1?001?11???????1????????0?001?10?0???????0??????????
?111???0???????????????000000000??????0000???0????

Jiangxisaurus_ganzhouensis
1??????????????????????11???????1?001????????11?????????????1??1??????111111??101?
1?0011?111????122??10?????????1???????1?1100?0?10????0???????????????????????????1
?001?10??2?????????????????0?00000?0?????????1?0??????????00??00???????????????????
??????????????????

Nankangia_jiangxiensis ??
????????????????????11101?2?????100?????????????2???????1???1??1??0?0011001?????????
0111011111111211001111????????1101??0???????1?00??11?????0?1???1????11???????01??
?11?0???0??0?00??????????????????????11????0?0???10

Shixinggia_oblita ??
???1?2?1??1????????????????????1
10000000?????1????1?????????????????????????01????????????????????????????????????
?????????????????????????????????????00?????0

Similicaudipteryx_yixianensis ??
??0?11?0?1?0??1?2???????1????????0?0
01?21110?????????0??0?12???????????????????00?????????????????????0??????????????
?????10?????0?????000?0000???????1???0??0??20

Wulatelong_gobiensis
11?2211?11?1?1???02121?111?1??1?111001??00??11???????????????1111???????????????1
01????1??11????12???????1???100????2??????????1??????1100?211111121???????000?01?
??????????0??????011????????????000????1????1?10?101????????????0??0??0???????0000
00??????1?????10????

Yulong_mini
10201011101101101111210101110l0?01000111010?111110110??0????????0?????111111121
22011001111111?12122?100??1????001?10??1??0000101?21010?110?10??0??0?1?0??11?0
01010?11001011?11000?100110??????0?0000010???????1??????1110?01000??00???00????
?000000000??????0???????????

Luoyanggia_liudianensis ??
?????????????????????000????????0?????????????2???????????????????????????????????
0?1??011????1?????????????????0???????????1?00???0??000???????????00????????1?????
?????0????10????????????????????????????????????

Ningyuansaurus_wangi
10?????????0???0?????????
????????????0?0??????00???????1???0???00?0?1?20??0?100?11000?0?1??????1?0??2?0???
0??????0???01000?????????????????0??10?????0??1????????????00000?????????????????
?????????????????

Protarchaeopteryx_robusta ?????0??
??????????????????????0????????0????????????0?0??????1????????1?????????1011?21111
?100?011?0???????????000120???????????00000?000??0??????????????00????????0??????
??????????00??0?0?????000000000??????????????????

Anomalopes_zhaoi ??
??
???????????????1110???000?0??00???????1???
??????????????????01?????00??1????0?111111001011

5. Oviraptorosaurian body size data

We compiled a dataset containing estimated body masses for all oviraptorosaurian dinosaurs for which a femoral length measurement was available, in order to investigate size evolution in Oviraptorosauria. Body masses were calculated using an empirical equation based on a statistical correlation between body mass and femoral length [3]. We aware that this equation probably produces results that are not as accurate as those generated by some recently developed empirical equations [4], but we nevertheless consider the equation we have chosen appropriate for our study given that length measurements are readily available.

Estimated body masses (kg)

Chirostenotes pergracilis 54.8

Microvenator celer 2.9

Citipati osmolskae 121.7

Khaan mckennai 12.1

Ingenia yanshini 24.0

*Conchoraptor gracilis** 10.0

Oviraptor philoceratops 51.0

*Rinchenia mongoliensis** 73.2

Caudipteryx zoui 5.2

*Incisivosaurus gauthieri** 8.5

Protarchaeopteryx robusta 2.7

Avimimus portentosus 10.9

*Heyuannia huangi** 19.5

Gigantoraptor erlianensis 3246.1

CM 78003 247.8

*Hagryphus giganteus** 179.6

Nomingia Gobiensis 41.8

*Machairasaurus leptonykus** 30.4

*Nemegtomaia barsboldi** 88.8

*Banji long** 2.8

3. Supplementary figures

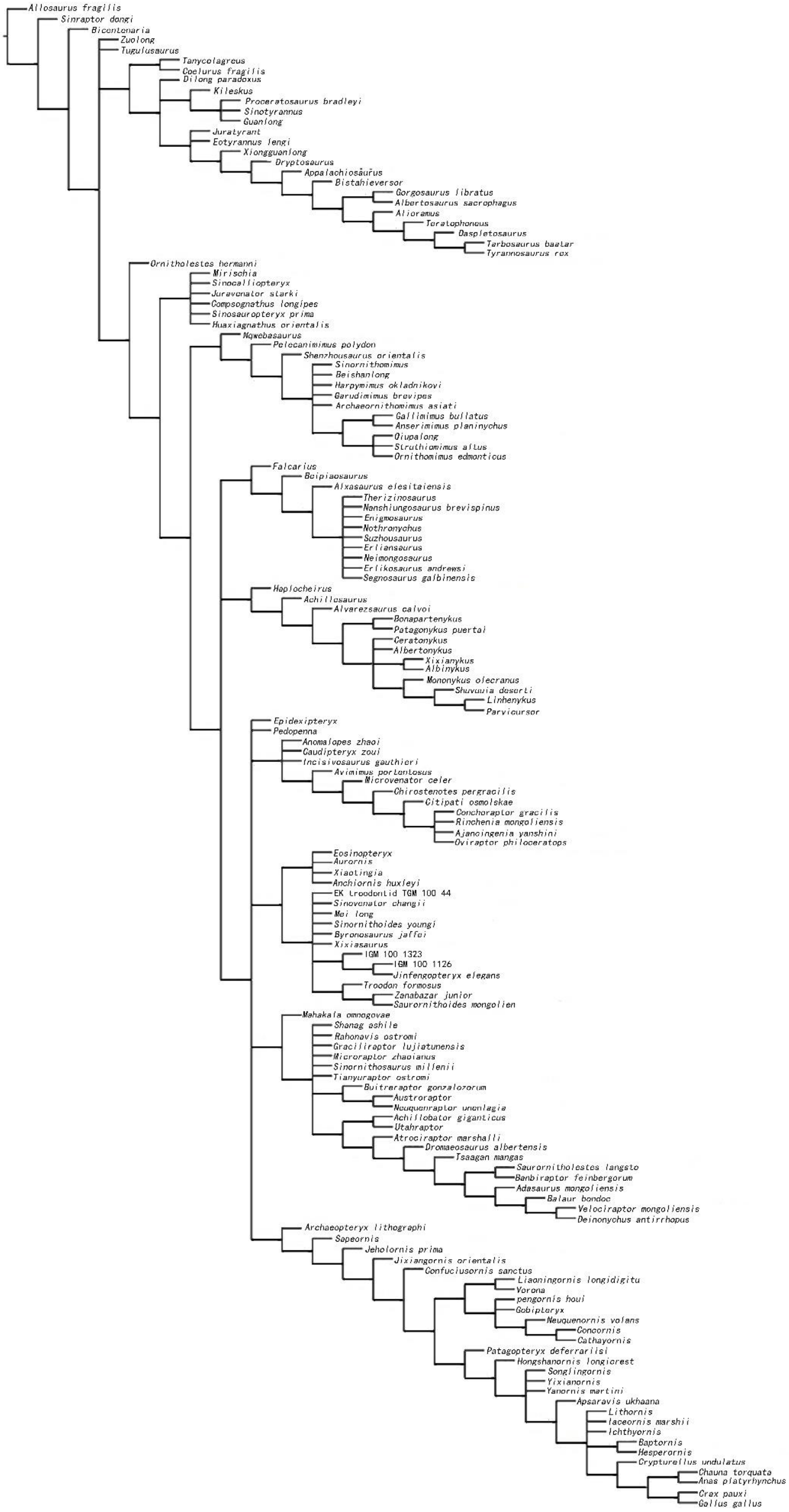

Figure S1. Strict consensus of 73 most parsimonious trees (length 3516, CI 0.311, RI 0.775) produced by our analysis of the modified Brusatte et al. Matrix.

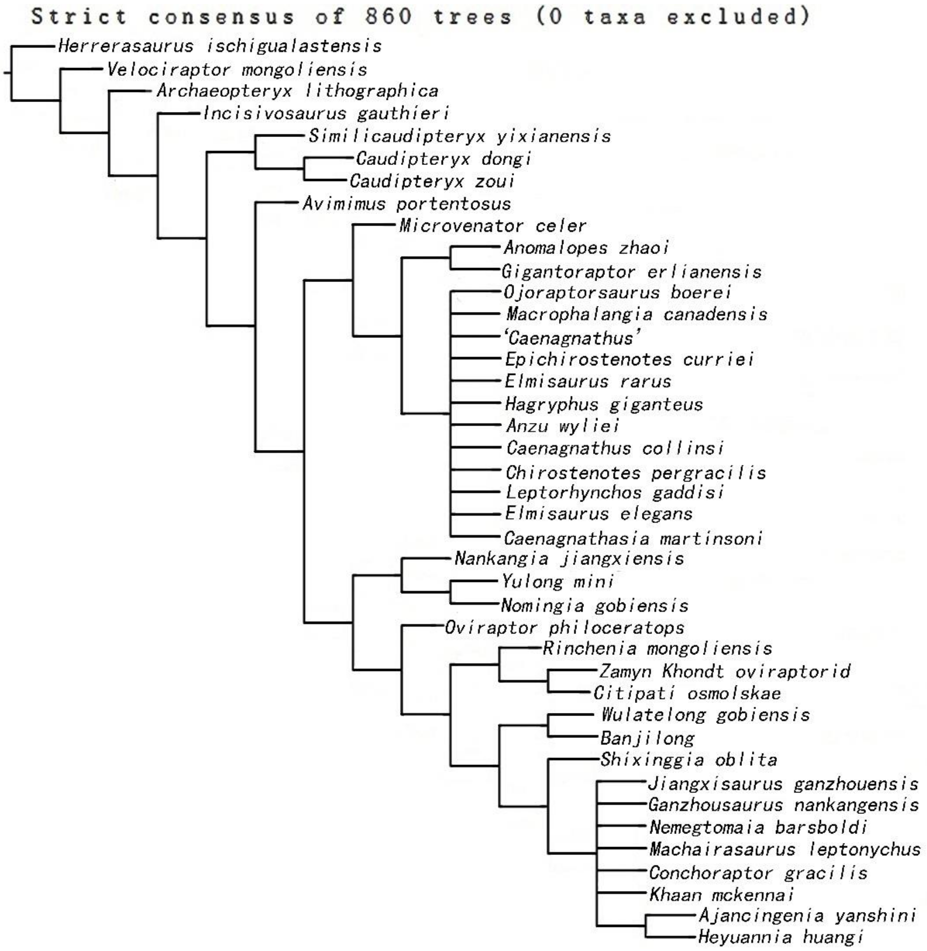

Figure S2. Strict consensus of 860 most parsimonious trees (length 552, CI 0.524, RI 0.687) produced by our analysis of the modified Lammana et al. Matrix

4. Supplementary references

1 Brusatte, S. L., Lloyd, G. T., Wang, S. C. & Norell, M. A. Gradual assembly of avian body plan culminated in rapid rates of evolution across the dinosaur-bird transition. *Current Biology* **24**, 2386-2392, doi:10.1016/j.cub.2014.08.034. (2014).

2 Lamanna, M. C., Sues, H. D. & Schachner, E. R. A new large-bodied oviraptorosauriantheropod dinosaur from the latest Cretaceous of western North America. *Plos One* **10**, e0125843 (2014).

3 Christiansen, P. & Fariña, R. A. Mass prediction in theropod dinosaurs. *Historical Biology* **16**, 85-92 (2004).

4 Campione, N. E., Evans, D. C., Brown, C. M. & Carrano, M. T. Body mass estimation in non-avian bipeds using a theoretical conversion to quadrupedal stylopodial proportions. *Methods in Ecology and Evolution* **5**, 913-923 (2014).
